# Assessing the governance of the health policy-making process using a new governance tool: the case of Lebanon

**DOI:** 10.1186/s12961-020-00557-1

**Published:** 2020-06-15

**Authors:** Rasha Hamra, Sameen Siddiqi, Emma Carmel, Walid Ammar

**Affiliations:** 1 Health Education Department, Ministry of Public Health, None, Lebanon; 2 Community Health Sciences Department, Aga Khan University, Karachi, Pakistan; 3 Social and Policy Sciences, University of Bath, Bath, United Kingdom; 4 Ministry of Public Health and Lebanese University, None, Lebanon

**Keywords:** Health system governance, Assessment tool, Health policy-making process, Good governance practices

## Abstract

**Background:**

In the international agenda, it has become common to assert that the assessment of health system governance using a practical tool is crucial. This approach can help us better understand how health systems are being steered as well as to identify gaps in the decision-making process and their causes. The authors developed a new assessment tool, the Health Policymaking Governance Guidance Tool (HP-GGT), that was designed to be conceptually sound and practical. This tool enables policy-makers and stakeholders to systematically review and assess health system governance at policy-making level. This article presents first use of the HP-GGT in Lebanon, together with generated results, recommendations, and discusses how these results improve governance practices when initiating new health policy formulation processes.

**Methods:**

The HP-GGT, which is a multidimensional structured tool, was used retrospectively to assess and review the process used to develop a new mental health strategy; this process was compared against consensus-based good governance principles, focusing on participation, transparency, accountability, information and responsiveness. The assessment was conducted through face-to-face interviews with 11 key informants who were involved in the development of the strategy.

**Results:**

The HP-GGT enabled policy-makers to reflect on their governance practices when developing a mental health strategy and was able to identify key areas of strengths and weaknesses using good governance practice checklists given by the questions. The insights generated from the assessment equipped the national policy-makers with a better understanding of the practice and meaning of policy-making governance. Identifying weaknesses to be addressed in future attempts to develop other national health policies helped in this regard. Using the tool also increased awareness of alternative good practices among policy-makers and stakeholders.

**Conclusions:**

Assessing a health policy formulation process from a governance perspective is essential for improved policy-making. The HP-GGT was able to provide a general overview and an in-depth assessment of a policy formulation process related to governance issues according to international good practices that should be applied while formulating health policies in any field. The HP-GGT was found to be a practical tool that was useful for policy-makers when used in Lebanon and awaits applications in other low- and middle-income countries to further show its validity and utility.

## Background

In 2007, the WHO began to use the term ‘health system governance’ (HSG). They defined it as “*ensuring strategic policy frameworks exist and are combined with effective oversight, coalition-building, provision of appropriate regulations and incentives, attention to system-design, and accountability*” [1]. HSG concerns “*how a policy is made rather than what policy is*” [2]. In other words, governance is about policy tools, techniques and methods as well as what ‘policy’ looks like in practice in terms of its goals and intentions [3, 4].

Policy-makers give more attention to governance issues related to how to decide to distribute resources, how to prioritise services and how to choose where to provide them by developing the policy content in terms of goals and intentions, and less attention to how to choose to organise and deliver services, whom to consult, and how to define and target social groups. Furthermore, they select indicators and benchmarks for policy evaluation, which is concerned with the process of how the policy was set and what tools and methods were used to do so [2]. To be effective, policy goals must be aligned with the institutional design and implementation strategies and be coherent with the concerns and interests of stakeholders. More specifically, high-quality governance in health policy-making is considered an important factor in shaping the improvement of health systems [1, 5], their functioning [6, 7], legitimacy [8] and outcomes [7, 9, 10]. With the agenda to provide universal health care in all countries [11], the development of effective and robust health policy-making processes is a particularly urgent and important challenge. This conceptual approach follows the normative claims about what constitutes ‘good’ HSG in policy-making circles.

The normative literature has generally proceeded by suggesting lists of principles and characteristics that constitute good governance in general, and these are supplemented by particular concerns for HSG [12–16]. There are overlaps in these lists, and they are variably supported by empirical studies of governance in practice. According to Siddiqi et al. [5], the most relevant principles/domains related to HSG are participation, transparency, accountability, the use of information, responsiveness, ethics, equity, efficiency and effectiveness, the rule of law, and strategic vision. This set of principles was based on the internationally recognised UNDP principles of good governance [12] and is one of the most comprehensive [17].

Policy-makers have a stake in improving governance at all levels to better steer and manage the health sector to meet their policy goals [18]. However, making high-quality health governance “*actionable*” [9] remains a challenge, given the abstract character of the principles. In low- and middle-income countries (LMICs), this challenge is exacerbated by conditions of resource scarcity and multiple urgent competing priorities for political and policy action, including conflicting development and donor agendas [19]. As Rashid et al. [20] suggest, ‘governance assessment’ needs specification to enable us to move from “*large and potentially vague*” principles to reach useful and actionable outcomes and recommendations. HSG remains a complex [21], sensitive and neglected topic in research [22]. HSG is the least understood among all the building blocks of the health system [5] and is difficult to measure, implement and evaluate, despite its importance.

In this article, we introduce a new governance tool (developed by the authors and accessible through Additional file 1 linked to this article) and explore the evidence of its practicality and feasibility in a case study application to review and assess the mental health strategy (MHS) development process in an LMIC – Lebanon. In addition to presenting the generated results and recommendations, we address how they were presented to and used by relevant policy-makers to improve their governance practices during the policy formulation process of a new health policy. The article will discuss how the new governance tool is different from the previous tools that were put in use as well as the limitations of using the tool and its findings.

### Context and scope of the HP-GGT: designing a rigorous and practical tool

In a systematic review, Pyone et al. [21] identified 16 HSG assessment frameworks. Only five of these frameworks have been used in practice and in a limited number of countries. In an attempt to overcome the challenge of making a specific enough assessment, the currently proposed tools in the literature focus on one aspect (e.g. corruption or transparency [23] or accountability [24]) or are sector/disease specific (e.g. pharmaceuticals [23] or HIV programmes [25]), limiting their relevance to policy-makers who need consistent tools that can be efficiently applied in a range of settings. Other tools have no sets of questions [16] or are too complicated to calculate an index, which is a quantitative measure that can allow comparisons within and between countries. This might be desirable for international organisations to rank countries accordingly. However, such an index might not be useful to policy-makers since it will not capture the full picture of governance practices, making such a tool impractical for policy-makers who need explicitly evidenced outcomes leading to actionable recommendations [17]. Most cannot assess change over time [5], limiting their usefulness in demonstrating improvements for policy-makers. The combination of the empirical complexity of HSG, the abstract and variable formulation of principles for governance, and the lack of practical tools hamper effective and robust HSG assessment [20, 25, 26]. Thus, there is a need for a tool that will enable practical assessment and suggest realistic interventions to improve the quality of governance processes.

Our Health Policymaking Governance Guidance Tool (HP-GGT) addresses these needs but cannot cover all gaps in assessing HSG. The HP-GGT is designed to be conceptually robust by drawing on an international consensus on health governance and covering a wide range of governance practices that are practical, efficient and easy to apply with actionable outcomes; furthermore, it is adaptable and applicable to diverse country and policy contexts. Starting from the work of Siddiqi et al. [5], we adopted a ‘conceptually rich’ approach to assessment based on multiple dimensions/principles [27] agreed upon in the international research and policy literature on normative governance perspectives.

The tool was developed following a systematic method that combined international research evidence with a Delphi consultation process. The Delphi consultations were conducted with 25 specialists in health governance and academic experts from 16 countries and international organisations over three rounds of online consultations. The first draft of the tool that was developed based on the available literature review contained a large range of questions that were organised around five key principles of HSG. The consultation round focused on determining what is relevant to the governance principles at the policy-making level, adding what is missing, rating the questions and then ranking them. The final draft after the Delphi consultations was subjected to face-to-face scrutiny by senior policy-makers from seven countries, at a workshop, following the RAND method [28, 29]. This final workshop focused on finalising the list of questions based on their importance and on the practicality, applicability and explicability of the recommendations to be generated from using the tool at the country level in order to enhance the tool’s relevance in real-world health policy-making environments. Details of the development of the tool were published as a doctoral thesis located in the University of Bath library [30].

The HP-GGT was developed to assess governance in national policy formulation and to serve as a mapping and guidance tool intended to benefit policy-makers at the national level. This tool focused on the development process of policies, not the content, as eventual policy outcomes are shaped from the outset by the process that was followed to formulate the policy in question [31]. If policies fail, the consequences might be substantial with respect to health outcomes and people’s trust in their governments. Therefore, following a good policy-making process should be an aim in itself for policy-makers who may not normally focus on the importance of the quality of the process [32].

For reasons of practicality, the authors decided to focus on an ‘acceptable’ number of principles – which was determined to be five– to generate a practical tool given the in-depth assessment it intended to offer; this number of principles was in line with other studies that have reported an average number of principles investigated of five [9, 16, 19]. The HP-GGT was organised around five of the ten components of Siddiqi’s framework, including participation, transparency, accountability, use of information and responsiveness given their particular importance in policy formulation. Stakeholder engagement, setting roles and accountability mechanisms, and establishing transparent goals and ideas [32] facilitate the alignment of stakeholders with policy goals, the coherence of institutional agendas, and clarity about the policy instruments and evaluation that are important to policy delivery. Stakeholder participation in the policy process can facilitate building the public trust in the process and in subsequent decisions [33]. Highlighting the importance of participation in LMICs is essential where hierarchal command-and-control types of governance face gaps and obstacles due to institutional weakness and/or a lack of authority. Accountability, transparency, responsiveness to the public’s needs and a consensus also contribute to the quality of public policy-making [32]. Evidence-based policy-making and a good understanding of different kinds of evidence are critical for policy formulation [31, 33] but the generation, use and evaluation of evidence can present a challenge for LMICs and their limited capacities (in terms of resources and knowledge needed) [34]. Furthermore, the tool was designed to capture the contextual factors and the policy environment that might affect the health policy-making process as well as the application of these five principles. These include political, economic, social, cultural, local, regional, national and international factors [35, 36]. These contextual factors might affect the policy-making process positively or negatively [37] and thus should be considered when evaluating policy processes and HSG as they reflect the reality of how HSG is applied.

The HP-GGT was designed to be a generic and flexible assessment tool that can assess any policy-making process retrospectively or be used prospectively to guide the policy formulation process (e.g. strategy on non-communicable diseases or maternity health services) in any country, particularly LMICs (where poor governance issues are common) [19], with minimal modifications, if needed, that will not affect its robustness.

### Structure of the HP-GGT

The HP-GGT is a structured questionnaire with two sections (Sections A & B). Section A has 53 closed-ended questions (CEQs) that evaluate the existence of the key structures, procedures, guidelines and legislation in relation to the policy. These attributes are those that would indicate the presence and application of different components/characteristics of governance principles. This section of the questionnaire is completed using a mix of documentary work and interviews. A long list of potential sub-answers to select from are examples of (and not limited to) the good practices identified from our tool-design research [30].

Section B has 30 open-ended questions (OEQs) that evaluate the ‘perceptions’ of the interviewees about good governance practices. These include questions on whether the procedures/legislations/policies identified by the CEQs are being implemented or enforced, and these questions facilitate an in-depth understanding of the policy-making processes in practice. The OEQs are designed to identify and elaborate the contextual factors affecting the development of a given policy. This section then provides systematically organised evidence that can be assessed to identify reasons for the gaps, problems or obstacles in relation to particular principles as well as how they might be addressed.

The tool covers the key features of the five principles that are summarised in Table 1 in order to attempt to assess those principles in-depth. Table 2 shows the domains of the five principles covered by the Siddiqi et al. [5] framework based on OEQs only to facilitate a comparison of the depth of their assessment with the HP-GGT.

**Table 1:** Major characteristics/domains of health system governance principles covered by the HP-GGT

Participation	Accountability	Transparency	Use and generation of information	Responsiveness
Types of participants	Components of accountability	Criteria for transparency	Generation, publication and dissemination of useful information	Elements of responsiveness
State actors	Answerability	Quality of data	Types of information	Respect for dignity
Health service providers	Sanctions	Speed of publishing data	Evidence based	Autonomy to participate in decisions
Public	Rewards	Ease of access	Financial resources	Confidentiality
Others	Enforcement	Mechanisms of transparency	Laws	Prompt attention
Representativeness	Types of accountability	Law to disclose	Values	Adequate basic health services
Organisations	Financial accountability	E-transparency	Factors affecting use of information	Communication
Themselves	Performance accountability	Freedom of press	External factors	Benefits of responsiveness
Benefits of participation	Political accountability	Written standard operating procedures and meeting minutes	Context	Human rights
Ownership	Benefits of accountability	Documentation of policies	Type of evidence	Improve wellbeing
Human rights	Control misuse and abuse	Benefits of transparency	Stakeholders and their relationship	Goal of health system performance
Knowledgeable people	Efficient use of resources	Increase public accountability	Benefits of generation and use of information	Direct outcome of governance
Democracy	Appropriate procedures	Increase public trust	Government encouragement and commitment for linking evidence to policy	Mechanisms to improve responsiveness
Negative impact of participation	Improved service delivery	Effective management	Making data generated at the service delivery level accessible to researchers	Institutional change
Time consuming	Actors in accountability	Reform component	Need for a mechanism to check funding sources of research to be used in policy-making	Enable participation (inclusion, voice and influence)
Conflict of interest	Policy-makers	Empower citizens	How research findings are adapted to local context	Media outlets (active and independent)
Costly	Private sector	Prerequisite for donors		Public polls, surveys
Barriers/facilitators of participation	Civil societies	Strategies to enhance transparency		Need measures of public preferences
Political will	Public	Institutional capacities and means to enhance it		Fair representation of all
Legal framework for participants to be involved in decision-making	Who is accountable to whom?	Publishing public service reports		Health policy should be assessed to ensure it meets population needs
Power struggle Financial resources	Mechanisms to foster accountability	Financial monitoring		
Context	Information system	Release of governments decisions		
Criteria for effective participation	Dissemination of information	Decisions related to priority-setting and financial allocation should be made public		
Consensus orientation	Watchdog organisations	Conflict of interest declaration by all stakeholders		
Transparency	Whistleblowing mechanisms	Information should be released in a predictable manner		
Available information	Types of sanctions			
Standard operating procedures	Legal sanctions			
Mechanisms to enhance participation	Regulatory sanctions			
Public inquires	Negative publicity			
Policy dialogue	Soft sanctions			
Citizen juries	Need to sign a contract/memorandum of understanding with stakeholders			
Assessments	Inform stakeholders that they will be held accountable before engaging them			
Roundtables	Public role in holding stakeholders accountable			
Contracts				
Committees				
Institutional, technical capacity and leadership to facilitate the participation process				
Gender consideration among participants				
Presence of dedicated resources to enable participation				
Using mechanisms to engage vulnerable groups				
Presence of a participatory body to oversee the implementation of policy

**Table 2:** Domains for assessing health system governance in Siddiqi’s framework, per principle^a^

Participation	Accountability	Transparency	Use of information	Responsiveness
Participation in decision-making process; stakeholder identification and voice	Internal and external accountability	Transparency in decision-making; transparency in the allocation of resources	Information generation, collection, analysis and dissemination	Response to population health needs; response to regional and local health needs

^a^The framework covers other domains related to the other principles that are not covered in our tool

## Methods

The assessment was conducted in Lebanon, which is classified as a middle-income developing country. The Lebanese health system showed resilience when facing the crisis related to the influx of approximately 1.5 million Syrian refugees into the small country [38]. The diversity of stakeholders has been identified as one key factor behind the resilience of the country [39]. Lebanon still struggles to improve the health system response to the crisis and to maintain the sustainability of the system; it was ranked 32rd out of 163 (and 1st in the Arab world) on the Bloomberg global health index for the healthiest countries by population in 2017 [40], given the limited resources and the political instability at the time of the assessment.

The mental health strategy (MHS) for Lebanon (2015–2020) [41] is given in Box 1, which provides a general overview of the MHS and its vision, mission and domains. This strategy was selected since it was already in place and was developed only 10 months before the assessment; it was launched officially in May 2015, and the assessment took place in March and April 2016. Thus, the assessment of the policy process in this case was performed retrospectively. The assessment focused on the development phase and not on the actual implementation of the strategy. Nevertheless, the assessment touched on some issues related to implementation that are assumed to be thought of during the development and should be part of the strategy, i.e. setting plans for the monitoring and evaluation process and issuing progress reports.

### Study design

The assessment included two parts. The data collection started with a general desk review to compile the background material and data collection from various documents in order to gain a clear understanding of the strategy developed, specifically in Lebanon, as well as to acquire a general perception of the health system and the mental health issues to comprehend the context. The inclusion criterion for documents was any official/national document about mental health (including laws, decrees, situation analysis and research studies) and the policy-making documents in Lebanon. The following documents were reviewed: the MHS (2015–2020) in both languages (English and Arabic), the relevant laws and policies related to mental health and those pertaining to drafting national policies in general, health statistics, media reports, newsletters, patient leaflets, and scientific publications. The ministry of health’s official website was also reviewed. The document review took around 2 weeks. The second part of the study included face-to-face interviews with the stakeholders and relevant policy actors using the HP-GGT questionnaire. Those individuals were asked to suggest any document that was not included in the desk review.

### Mapping of all stakeholders for interviews

We conducted a mapping of all stakeholders who were directly involved in the formulation process of the strategy to identify our potential key informants (KIs). The identified KIs were deemed to be knowledgeable about the policy formulation of the MHS of Lebanon and were directly involved (state and non-state actors). Thus, purposeful sampling was employed to ensure the selection of ‘information rich’ KIs using snowball/chain sampling [42]. The multiplicity of stakeholders (professional associations, NGOs, academia and international organisations) in Lebanon is of utmost importance in policy-making due to the multi-denominational nature and fragile government institutions in the country.

### Approaching potential KIs

The KIs involved in the strategy development were categorised into eight categories (Table 3). A total of 20 potential KIs were contacted by email with an invitation to participate in the assessment. The KIs were given the choice to sit two interviews: one interview for each of the two sections of the tool starting with the CEQs. The KIs were informed that each interview might require approximately 50 minutes, and they were given the option to have one interview to cover both sets of questions. Longer interviews were conducted on more than one occasion [43]; other studies suggest that interviews lasting 50 to 90 minutes are acceptable [44]. The suggested time lapse between the two interviews was set as 1–2 weeks at most. The intervening time between the two interviews actually offered both the assessor and KIs an opportunity to reflect and contribute to the learning from the tool.

**Table 3:** Summary of key informants identified, contacted and interviewed

Key informant group	Number identified and contacted	Number interviewed
UN agencies	2	2
Local NGOs	2	1
International NGOs	3	1
Universities	3	2
Professional associations	2	1
Mental health units and hospitals	2	1
Governmental agencies other than Ministry of Health	2	0
Mental Health Programme Team – Ministry of Health	4	3
Total	20	11

Of the 20 KIs contacted, 11 responded positively to the invitation, with a response rate of 55%. None of the contacted KIs from other governmental agencies, such as the Ministry of Social Affairs and the Ministry of Justice, responded to the invitation email; thus, this category was not included in the assessment. This lack of response could be due to difficulties in approaching governmental staff by email and not being interested in such studies due to the lack of interest in the topic or the lack of awareness about its importance. Out of the 11 interviewed KIs, 3 (27.3%) decided to sit for the two sets of interviews in one session, 5 (45.4%) opted for two different times, and 3 (27.3%) did not perform the second interview (due to a tight schedule).

Hence, 11 KIs answered the CEQs, while only 8 answered the OEQs. All the interviews were conducted over a 2-month period. All questions were asked in relation to the aforementioned MHS that had been recently developed.

Thematic saturation, that is, having no further useful information from KIs [45], was reached after seven KIs had been interviewed; however, the recruitment process was continued since the target was to include all categories of stakeholders in the assessment to ensure the collection of as many perspectives as possible.

### Analysis of the assessment findings

Descriptive statistics were generated for the CEQs, which merely entailed a simple counting of the responses (yes, no, don’t know, in process and not applicable). Based on the results of the CEQ section and to prepare a useful brief of findings for policy-makers, a traffic light symbol summary (usually used by the WHO to present findings to policy-makers [46]) for each of the five principles (to highlight what each principle means in practical terms as a way to broaden the knowledge of policy-makers) was used. Red indicates that a certain practice does not exist or is not practised; yellow means that a certain process is either in progress or exists but is not practised or exists but stakeholders are not aware of it; and green means that a certain practice is in place, practised and well known to all. These colours give signals to policy-makers to work on turning the yellow and red into green and maintaining the green. Thus, traffic light lists can be used to evaluate progress or changes over time (using change to traffic light colours when assessment is repeated to document this change). The traffic light lists were based on the CEQs and their sub-answers; the questions and sub-answers were listed as they appeared in the tool and a colour was assigned to each based on the answers of the KIs (which were all consistent, and only a few did not know an answer, resulting in ‘don’t know’ being assigned). The traffic light summaries are attached and shown in detail in Additional file 2A.

Thematic analysis was also conducted for the OEQs after the transcription of the interviews to come up with the main themes that were common between KIs [47]. A Strengths, Weaknesses, Opportunities, Threats (SWOT) analysis on the formulation process of the strategy was prepared based on the themes that emerged from the OEQ section only and was also presented to policy-makers to complement the findings of the traffic light lists (Table 3).

The results from the CEQs and OEQs were compared against each other and against the findings from the desk review and were all triangulated to check if there were any discrepancies.

## Results

The purpose of this section is to highlight the actionable findings that were translated into practical recommendations, how they were presented to policy-makers and how these findings, as such, initiated change in the governance process that was followed based on the implemented recommendations (presented below). We will offer a general interpretation of the findings (not an in-depth analysis) since the scope of this article is to present the feasibility and the practicality of using the HP-GGT as an assessment policy tool and to demonstrate how presenting the results in a simplified manner to policy-makers encouraged action from their side.

Using the HP-GGT allowed the evaluation of the application of the governance principles during the development of the MHS. The general findings from the CEQ section are presented in the traffic light tables, where they highlight that the accountability and responsiveness principles were more red than green, participation was mostly green and transparency was mostly yellow. These findings were analysed and classified based on the presence or absence of the characteristics/domains of the principles that were covered by the tool (Table 1). Thus, we further identified the gaps and the strengths in the application of the principles according to their specific characteristics. See the summary table in Additional file 2B.

In this section, we will highlight the most important findings (based on the table in Additional file 2B, which is a reflection of the traffic light lists that were presented to policy-makers), most of which had practical implications for the practice of policy-makers at the ministry level, and include the following: in terms of ‘participation’, the assessment showed that the policy formulation process was inclusive of almost all relevant stakeholders (despite there being no legal requirement to include various stakeholders in health policy-making) except for parliamentary members and patient groups. Including parliamentary members is essential to having coordinated efforts to lobby for the enforcement of laws and passing new ones that are lacking (based on the findings of the accountability section). Excluding patient groups might be one of the reasons for the poor responsiveness of the strategy to the beneficiaries’ needs, as was reflected in the traffic light results. The working group responsible for the development of the strategy was not officially formed by a ministerial decree or any relevant mechanism (no formal authority was given to the working group) and no specifications for the mandate or the qualifications of the members were set. Thus, the process followed for the development of the strategy was not formal. This might be one of the reasons for the lack of accountability mechanisms. Meeting minutes were recorded but not published. The public and other (non-participating) interested stakeholders did not have the chance to give their opinions, which resulted in limiting the participatory process to actors who were invited to participate only. The roles and responsibilities for the implementation plans were not set within the strategy and a participatory body was not appointed to oversee the implementation process. There was simply a lack of coordination once the strategy was set, and stakeholders were not informed about the implementation progress. This highlights the need and importance to plan for the implementation process during the development phase.

The identified barriers to participation included stakeholders having different agendas, a lack of incentives, difficulties in finding an appropriate time and place for everyone to meet, and a lack of official obligations and assigned responsibilities since the working group was not a legal entity and worked on a voluntary basis based on their free time. On the other hand, there was a high level of commitment and dedication from the leadership within the ministry to develop a MHS for the first time in the country, funding for its implementation was available (through donors, but this is not sustainable since it is not done through the Lebanese government), all stakeholders were motivated and knew each other, and the final decisions were made by consensus. All of these indicated that there was a strong participatory approach led by the ministry.

As for ‘accountability’, there were no formal mechanisms to hold public officials and non-state stakeholders involved in the policy development accountable (as they were working through non-formal mechanism), and the public did not have the chance to hold stakeholders accountable for the decisions made on their behalf. There were no standards or monitoring nor any enforcement mechanisms for the implementation (since these were not planned during the strategy development phase) and there was a lack of sanctions to impose in the case of a violation. There were no whistle blowing or watchdog protection mechanisms in place to ensure that the strategy would meet the legitimate public needs, and no independent monitoring and evaluation was planned. All of those accountability mechanisms were not embedded in the MHS document. The current mental health law was also not enforced, though its enforcement is essential for strategy implementation. There were plans to generate key performance indicators, but the data collection process did not start at the time of the assessment since it might have been too early to do so since the assessment was only 10 months after the strategy was launched. This highlights the importance of setting plans for monitoring and evaluation as early as possible and to be part of the developed strategy. Based on the answers of KIs, the media were not well informed about the MHS. Well informed media are needed to have an additional tool to strengthen the accountability mechanisms that are already weak, as was shown in the traffic light study due to the weak implementation of the main components of the principle in practice.

As for the ‘transparency’, the mental health programme team believed that the process they followed was transparent according to one KI, but the non-state stakeholders believed the opposite. The tool was able to detect such findings by giving the opportunity to policy-makers (within the Ministry of Health; MoH) and other stakeholders to reflect on the specific issues of governance during the formulation process in which they were involved. The priority-setting was not transparent, no clear justifications for the goals were set, and resource allocation decisions were not made public to all. The stakeholders who were involved in the policy development were not asked to declare whether they had any conflict of interest or to sign any agreement on the scope of work to be carried out; thus, their participation had no assigned responsibilities. The relevant implementation decisions/operational plans (e.g. who will do what and when) were not shared or published, which resulted in stakeholders feeling that they were excluded from the policy implementation phase once the strategy was set in place. This is crucial since the MHS was meant to be a national strategy (as reflected in Box 1) and not a ministry strategy; thus, implementation required the efforts of all relevant actors in the field. On the other hand, the strategy was published on the ministry website, it was easy to locate, and the final document was published in English and Arabic (the national language in Lebanon). The strategy was also announced using mass media, newsletters and emails. The strategy document was user friendly and included the magnitude of the problem, objectives, evidence used and general timeframe for implementation (5 years). The access to information law was waiting to be passed by the Lebanese parliament and work to set relevant laws to promote electronic government services to improve the public access to government information and services was under study by the government (these are beyond the ministry’s capacity). In parallel, the ministry and the mental health team were working on strengthening the transparency mechanism by working on publishing and disseminating progress and policy evaluation reports in a periodic manner.

Regarding the ‘use of information’, the ministry did not allocate any budget for relevant local research, there was neither a specialised unit nor a specific registry at the ministry to address research analysis for policy-making, and the raw data generated at the service delivery level were not available for researchers. This situation reflects the weak institutionalisation of the use of evidence at the ministry level, which requires the availability of human and financial resources that were not available at the time of the assessment. The strategy was developed based on scientific evidence currently found abroad, though it is reliable, of good quality and locally appropriate. However, financial information and public opinion were not considered during the development of the strategy and, thus, some services were promised without an accurate estimation of the costs or appropriateness for the people’s needs.

Regarding the ‘responsiveness of the public needs’, the strategy set patients’ rights but not their responsibilities and did not include plans to conduct patient satisfaction surveys at the service delivery level. The strategy did not explicitly state a benefit package to be provided, how referrals will take place from one level of care to another or set a reasonable timeframe to provide the needed services. This might reflect the poor responsiveness of the strategy during the planning and implementation phases since beneficiaries are not informed about how, when and where they can access mental health services, and these issues reflect the poor communication and transparency. However, the strategy stated that all people living in Lebanon (including refugees) will have access to quality services, including disadvantaged/vulnerable groups (with defining those groups), and the services to be provided will respect the confidentiality and dignity of the mental health patients yet there are no set mechanisms to ensure those obligations. The full findings are available upon request from the authors. All of these findings are reflected in the traffic light summary.

SWOT analysis was conducted to complement the findings presented in the traffic light study (and not to be inclusive to avoid repetition that might burden policy-makers) since it was based on the themes that emerged from the OEQs, which gave some details about the contextual factors that affected the governance process of the MHS development that reflected the opportunities, as well as the challenges/threats, that were faced and the reasons for the gaps that were identified (Table 4). The OEQs within the tool enabled the identification of these opportunities and challenges that need to be considered by policy-makers since they might affect the implementation of the strategy itself or the development of future national policies. These factors were the political will at the ministry level that supported the development of the MHS for the first time and were thus committed to supporting the provision of mental health services and the implementation of the strategy, financial factors (availability of funding earmarked for mental health issues yet from donors and not from the government, which will affect sustainability of planned activities), cultural issues (mental health is a taboo topic in Lebanon), and a window of opportunity to work on the MHS (i.e. Syrian refugee crisis in Lebanon and interest of donors working in humanitarian settings to fund such activities). It was found that the institutionalisation of governance practices would require human and financial resources that were not available at the time of the assessment, and this affected which recommendations were prioritised for immediate implementation (ones that needed the fewest possible resources). The strengths and weaknesses that emerged from the OEQs were triangulated with the findings of the CEQs and were found to be consistent. These findings are indicators of good governance practices that need to build on strengths and address the gaps in future policy-making processes. It is worth mentioning that most of the governance issues explored by the assessment were not reported in the documents reviewed, thus setting the need to document the policy development process from the governance perspective.

**Table 4:** SWOT analysis on formulation process

Strengths	Weaknesses
• There is a new national programme with a motivated team	• No formal national committee or working group was formed for the development of the national strategy; no written mandate for roles and responsibilities
• Commitment of the Ministry of Health (MoH)/national programme to coordinate with all and involve all	• No structured process was followed; nothing was documented
• Leadership of the MoH and the national programme were key to success	• No follow-up was conducted with stakeholders regarding implementation plans and monitoring and evaluation
• Mental health is now a priority for the MoH	• No transparency with regard to implementation plans or progress reports and roles and responsibilities were not defined
• The Mental Health Strategy is in place and serves as a guiding roadmap	• Some stakeholders were not involved (see traffic light)
	• Public was not informed about the strategy
	• Accountability mechanisms were weak/almost absent; no standards, sanctions or enforcement mechanisms were set
Opportunities	Threats/Challenges
• Availability of funding by donors	• Sustainability unclear once the funds are exhausted
• All stakeholders were motivated to be involved	• Lack of strategic planning of next steps and resource mobilisation
• Political commitment positively influenced the strategy to include all people, not just Lebanese and vulnerable groups; plans exist to encourage the establishment of patient support groups	• Accountability is a cultural issue that is related to what is right and wrong and remains a vague concept
• Technical support was provided by international agencies as well as international and local experts	• Need to pass the amendments on the current law and enforcement
	• Receiving funding from the government
	• Governance requires institutional capacity, appropriate structure and sustainable financial resources

Based on the findings and the SWOT analysis, a list of recommendations was generated to address the gaps in future policy formulation processes by implementing good governance practices and, if applied, this might improve the policy formulation processes aiming for better policies (see Table 5, left column, for some of the most important recommendations).

**Table 5:** Recommendations to policy-makers for future policy formulation process and what was implemented

Recommendations	Implemented recommendation
• National committees/working groups responsible for policy formulation should be officially/formally formulated by a ministerial decree or by a similar mechanism	• A national committee was formed by a ministerial decree to work on the development of the national strategy on substance use and abuse
• Mandate for work, including roles and responsibilities and timeframe, needs to be set and documented	• The decree set a timeline for the committees’ work and specified the general role of the committee
• The inclusion of the public (patients and beneficiaries) and parliamentary members, if possible, is recommended	• The committee included patient groups
• Involve media in the policy formulation process to sensitise them regarding issues related to the policy concerned; training the media on tackling health issues is recommended	• A media tool kit was developed to sensitise the media on mental health as well as on addiction issues; training on the kit is planned
• Document meeting minutes and share them with all stakeholders	• Meeting minutes are being documented and shared by email for feedback and approval
• Need to form a participatory body to oversee the implementation of the strategy; ensuring participation throughout the policy-making cycle is crucial for good governance	• The draft strategy on drugs and addiction was published on the Ministry of Health (MoH) website for 2 weeks for public feedback
• Operational plans/implementation plans should be published and shared with all	• The Mental Health Programme started signing memoranda of understanding with relevant stakeholders for implementation of the Mental Health Strategy (MHS)
• The public (including scientific entities, academia, media and the lay public) need to be informed regarding draft policies/strategies and should be given the chance to forward feedback and comments	• An independent body was recruited to conduct a mid-term evaluation of the implementation of the MHS
• All participants should sign memoranda of understanding and conflict of interest declarations before being engaged in policy formulation	• A hotline was activated for all kinds of complaints to the MoH, including issues related to MHS, but results of the complains are not published
• The MoH/national programme should set formal accountability mechanisms to hold various stakeholders accountable during the formulation and implementation phases	• Independent monitoring and evaluation (M&E) results were shared in a big meeting with all stakeholders that were involved in the formulation and implementation of the MHS and they appreciated the transparency and the efforts to include all; the level of trust was increased; results not published yet
• The MoH/national programme should work on setting standards and sanctions as well as incentives	• Set a collaboration mechanism to set a priority list for research with academic institutions in the country
• The MoH/national programme should set in place a complaints system and publish the results of complaints investigations	• A mental health registry was established to register cases as well as map services provided by all stakeholders
• The MoH/national programme should disseminate progress reports as well as M&E reports and other relevant documents to all stakeholders and publish these on their websites	• A benefit package was set at the primary healthcare centres
• The MoH/national programme should develop and publish financial reports on the sources of funding as well as how funds were allocated and spent; financial information should be taken into account when formulating a policy	
• Collaboration to conduct local research on relevant issues and funding, if possible	
• Needs assessment targeting the public should be conducted both before the formulation of health policies and after implementation to assess responsiveness of the policy to public needs as well as to the services provided as a part of the policy	
• There is a need to have a specialised unit/staff for research analysis and for policy-making	
• A national mental health registry is needed	
• A benefit package should be clearly stated within a policy/strategy with a timeframe to provide services as well as setting a referral system, so the patients/service users know what to expect	

The traffic light represents a long list of processes that were present/absent from the policy formulation process when the MHS was developed. This list was given to policy-makers to let them decide on the ‘key’ issues that they would like to prioritise to work on in future policy formulation processes. This was deliberately done for policy-makers to take the lead and feel ownership of setting their list of governance priorities and improving their governance practices. For the authors, all identified gaps were of equal importance and thus were not given different weights.

The traffic light, SWOT analysis and recommendations of the assessment were presented to the national mental health team for their reflection and feedback and to set their priorities for future work. One high-level official commented on the findings: “*the tool depicts reality of how things were done. Recommendations are very useful, and we have already started using them for other strategies. This exercise has been very useful for us*”. The mental health team implemented some of the recommendations while developing a new national strategy related to substance abuse (see Table 5, right column, for the recommendations that have been implemented to date).

## Discussion

The implemented recommendations were probably the most practical to the policy-makers/mental health team to implement since they greatly impacted the relevant stakeholders and the public. The suggested recommendations presented to these individuals enabled them to take specific steps that were practical and easy to implement. These individuals reflected on their efforts to strengthen the participatory process by formalising it (by issuing a relevant ministerial decree) and expanding the pool of participants (including patient support groups) in the decision-making consultation process in addition to making the effort to publish the draft strategy on the official website to open the door for feedback from anyone who was interested in contributing. Additionally, the mental health team decided to make an effort to increase the transparency by increasing the amount of documentation and the dissemination of the relevant documents to gain the trust of the relevant stakeholders and the public.

Strengthening accountability mechanisms were given attention as well by having an independent body conduct mid-term evaluations of the strategy evaluation by 2019. The roles and responsibilities for the relevant committee were written and communicated to other stakeholders; this was done to hold the members of the committee accountable when needed. Sensitising the media on issues related to mental health and substance abuse was another effort done by the mental health team to improve participation and was an added tool for accountability by monitoring the implementation. Collaborative efforts were established with academia to promote the use of information in policy-making and planning by establishing a national registry for mental health disorders and setting national priorities for research in the field so that efforts were in line to fill the gaps and generate the needed evidence.

Setting a benefit package specific to mental health services at the primary health centre level and announcing it widely to the public was an extra step towards being more responsive to the needs of the public in practice in addition to being responsive inside the strategy document. Thus, using the HP-GGT in Lebanon demonstrated that the mental health team at the MoH appreciated the findings of the assessment, as they wanted to improve their governance practices by taking concrete steps to produce more evidence-based policies that are responsive to the needs of the public in a transparent and accountable manner by involving all relevant stakeholders to gain the trust of all and to improve the decision-making process; this is in terms of the presented results and the recommendations.

Regarding the HP-GGT itself, it was found to be different from other existing HSG assessment tools, as demonstrated by the case study of the MHS in Lebanon. The difference is in terms of, first and at most, the in-depth assessment of the good governance practices in policy formulation it provides by exploring the five principles and their characteristics by translating those into actionable processes that are simplified and reflected in the questions. In turn, these questions constitute non-exhaustive but detailed examples of good practices (for example, listing all stakeholders for an inclusive process). These characteristics may not be exhaustive, but they offer a comprehensive list based on international research and Delphi consultation that could be expanded or revised based on the lessons learned from other countries. By providing this clear overarching, five-principle framework, such revisions will be easy to accommodate.

Second, the tool assesses the governance process for future improvements, and enables assessing change over time by using traffic light summaries and tracking the colour changes. Despite that, tracking change over time is done in a descriptive manner since it is done by repeating the assessment at successive time points to evaluate what practices/processes have changed and is reflected by the change in colour in the traffic light. These results will enable policy-makers to reflect on their practices and highlight ‘good’ practices using the list of questions of the tool as a checklist. Thus, the tool proposes interventions that can be used to improve future policy-making processes (i.e. if there is no specialised unit within the MoH that addresses research analysis for policy-making, a unit with qualified staff should be established). In our study, the tool also enhanced the sense of responsibility among stakeholders regarding their own participation to improve HSG quality.

Third, the HP-GGT is an empirically flexible and practical tool. It can be used as an assessment tool to evaluate a policy-making process retrospectively or it can be used prospectively to facilitate and guide the policy formulation process, facilitating the analysis of the governance processes for the health policy formulation, including the contextual factors. The tool is also flexible since the unit of analysis can be adjusted. In this assessment, it was the MoH who was responsible for developing national policies at the central level. In other countries, the unit of analysis might be a responsible independent body or a devolved sub-national authority such as in Pakistan. The tool can also assess other types of national health policies in any country with some adjustments if needed. The HP-GGT does not require many resources or special expertise to be conducted, and it requires a relatively short period of time for data collection and analysis (all in all, it took 3 months). Thus, using the tool was found to be practical in terms of the resources and time needed and its ease of use. The length of the interviews was not an issue since all KIs found the topic and the questions of interest to them as well since they were informed beforehand about the time needed for the interviews and the timing was set accordingly. Some of the feedback that was received from different KIs concerned the length of the tool: “*It needs time to think about the answers, but it is challenging and enjoyable*”, “*Although long, it is an important tool and worth the time*” and “*I enjoyed it and did not notice the time and it is worth it*”.

As for the desk review approach followed for the case of mental health in Lebanon, it was acceptable since there were a limited number of relevant documents that were identified and they did not require much time to be identified and reviewed. This approach might also be reasonable in other LMICs due to the limited number of publications related to policy-making, policy analysis and health system governance as per the literature [22, 33]; this possibility is to be further explored with more case studies.

The HP-GGT should be tested in other countries with different policy types, including those less developed than Lebanon, those with more complex health systems, and those with other types of policies, such as universal health coverage policies, that are broader and more complex. The tool should be applied in a range of other settings to test its usefulness to other policy-makers, its practically in different countries, and its reliability with different policy processes.

Our approach has some limitations. The tool, in order to be specific and practical, focused on policy formulation processes although, in practice, it is difficult to separate policy formulation from implementation due to the overlap of these steps and the actors involved. However, it is not uncommon to focus on a particular stage of the policy process rather than on the whole policy cycle [48], and by choosing to narrow our focus here, we were able to design a tool that could be consistently applied in a range of settings according to our aims. Concentrating on a single stage of the policy cycle (formulation) was found to be useful for improving the understanding of that stage as a prerequisite to other stages and its influence on policy outcomes [31, 49]. Another similar tool could be used to assess the implementation of national policies and, thus, would be considered an evaluation tool.

The HP-GGT tool operationalised five governance principles only, which meant that it was focused, realistic and more adaptable to developing countries. According to Bovaird [50], “*all* good governance *principles are important; however, they are not all equally important to all stakeholders in all contexts*”. Other principles, such as the rule of law, equity and efficiency, have specific evaluation tools that exist and are commonly used.

The limitations of the results generated by the tool include the following: first, any governance assessment generates partial findings since they cannot capture all governance problems at the chosen level within the health sector; thus, the results might reflect a partial reality of health governance but provide an in-depth analysis about whether the five principles are applied. Second, if there are actual conflicts that might exist between different strong stakeholders pushing for ‘good governance’ against inequity, corruption, ignorance of evidence, etc., KIs might not open up to reveal such conflicts if no trust/link is established with the person conducting the assessment; thus, the questions might not elicit genuine answers from stakeholders. Building trust between the KIs and the assessor is important. Furthermore, the tool focuses on assessing the existence of a formal structure, strategies and practices within the developed strategy to ensure good governance, but it cannot predict whether those will be implemented/practised or enforced. For example, under ‘responsiveness’, it revealed that “*the strategy ensures that all will have access to quality services including disadvantaged/vulnerable groups and ensures that all health services related to mental health will respect the confidentiality and dignity of all*”, though there is no way to know if this will be implemented or not. However, there is an importance of this being documented in an official strategy with good accountability mechanisms in place since policy-makers will be held accountable by other stakeholders, the public and the media to fulfil this promise. Policy-makers need to be aware of/highlight this commitment as part of being responsive to the needs of the public.

The eventual contribution of the tool will also depend on the political, institutional and technical issues affecting the implementation of recommendations [51, 52]. Applying all recommendations while developing/formulating a new national policy/strategy might cause a delay in the formulation process if the institutional capacity is not in place [53] or if there is resistance from others to apply good governance practices [54, 55]. In the end, policy-makers must decide both what to prioritise and how to prioritise according to the context in which they operate. For example, publishing relevant and updated information on a regular basis on the MoH website is much easier than passing a law that allows access to information; nevertheless, both are essential and needed. Aiming for ‘good enough’ governance is a more realistic goal than strict lists of idealised ‘good governance’ attributes [54]. The results generated from applying the tool are specific to a specific policy process in a specific country, but the recommendations can be generalised to any other health policy process in that specific country and this was evident in the implemented recommendations with the substance abuse strategy. Although the tool is meant to be flexible and generic, it is developed to better fit developing countries that are challenged the most by integrating good governance principles in their practices since they are frequently identified as those where current governance systems and practices at all levels affect their development level [19].

Future research should focus on the other principles and on different phases of the policy-making cycle such as implementation. Another avenue for investigation would be to expand the list of good practice examples from other countries and explore the good practices that are suitable for different settings in detail. Applying the tool in various countries will help in generating and documenting what different countries are doing to improve their HSG at the policy-making level, which is recommended for “*collective action across countries*” to improve governance [26].

## Conclusions

Assessing governance at the policy-making level can be used to initiate change, identify weaknesses, provide insights and open up thinking about potential solutions and thus facilitate the improvement of the policy process [56], as was evident from using the HP-GGT in Lebanon. It is hoped that governance at the health policy-making formulation level will encourage the application of good governance principles that will help strengthen both the institutional capacity and stewardship role of MoH/health authorities in LMICs and improve the policy-making process. Thus, assessing HSG is a first step towards benchmarking health governance good practices at the policy-making level in line with the international development agenda. This required a new and different approach to assess and provide guidance to policy-makers using a practical assessment tool – the HP-GGT. This tool can be used as an entry point for reflection since reflection is necessary for policy-makers, yet it is rarely exercised [32]. The HP-GGT is an informative tool to evaluate ways to improve the policy-making process, regardless of the content of the policy or final outcome of the implementation. While this tool cannot offer solutions to control the context in which the policy formulation takes place, it can highlight the opportunities and challenges that might be imposed on the formulation process and give policy-makers ownership over the priority changes they want to implement in their governance practices. This article will make the HP-GGT available (through additional links provided) to any policy-makers, researchers or stakeholders who are interested in assessing HSG.

The tool is not an end by itself but rather the beginning of a process to improve the understanding, importance and application of health governance in practice. Improving governance is a complicated and ongoing process that would require institutional changes at many levels; there is no universal solution for all governance problems [57], and the required changes cannot happen all at once [54]. Moreover, improving the governance quality involves changes to long-standing practices, strategies, interests, cultural habits and social norms; thus, the change process should be gradual. Siddiqi et al. [5] stated that the “*road to good governance in health is long and uneven*”. We would like to consider this work a starting point on this road and an opportunity to open a concrete dialogue with policy-makers.

Box 1 Overview of Mental Health Strategy vision, mission and domains

- **Scope of strategy and guiding principles**

It is a national strategy that set a framework for all relevant stakeholders working on mental health in Lebanon and not just for the Ministry of Health. It was the first time that mental health issues are tackled at national level. The development of the strategy was marked as an important milestone for mental health reform, especially considering that mental health and substance use disorders are prevalent and at the top of public health priorities.

The main goals of the strategy are based on governance principles of participation, responsiveness and use of information as evidence.

- **Vision**

All people living in Lebanon will have the opportunity to enjoy the best possible mental health and wellbeing.

- **Mission**

To ensure the development of sustainable mental health system that guarantees the provision and universal accessibility of high quality mental health curative and preventive services through a cost-effective, evidence-based and multidisciplinary approach, with an emphasis on community involvement, continuum of care, human rights and cultural relevance.

The goals and domains of the action of the strategy are in line with the WHO Global action Plan for Mental Health (2013–2020).

- **Goals and Domains**

The strategy covers five domains:

- Domain 1. Strengthening effective leadership and governance for mental health.
- Domain 2. Providing comprehensive, integrated and responsive mental health and social care services in community-based settings for all populations, especially the needs of specific vulnerable groups (Domain 5).
- Domain 3. Implementing key promotion and prevention activities for mental health and substance use disorders.
- Domain 4. Obtaining evidence-based knowledge to inform mental health policy and service development through an operational health information system and coordinated national research practice

## Supplementary information

**Additional file 1.** Health Policymaking-Governance Guidance Tool (HP-GGT), Final HP-GGT tool.

**Additional file 2.** Traffic lights summary and summary of findings by principle and characteristics; data presentation of the pilot results that was submitted to policy-makers.

## Data Availability

The datasets used and/or analysed during the current study are available from the corresponding author on reasonable request.

## Abbreviations

CEQ: Closed-ended questions
HP-GGT: Health Policymaking Governance Guidance Tool
HSG: Health system governance
KIs: Key informants
LMICs: Low- to middle-income countries
MHS: Mental Health Strategy
MoH: Ministry of Health
OEQ: Open-ended questions
SWOT: Strengths, Weaknesses, Opportunities, Threats
