# Supplementary material for: Assessing the governance of the health policy-making process using a new governance tool: the case of Lebanon
Source: Health Res Policy Syst. 2020 Jun 15;18:66. doi: 10.1186/s12961-020-00557-1 (PMC7294613; doi:10.1186/s12961-020-00557-1)
Supplement: Supplementary file 1 — Additional file 1. Health Policymaking-Governance Guidance Tool (HP-GGT), Final HP-GGT tool. [file 12961_2020_557_MOESM1_ESM.docx]

**Additional File 1: Health Policymaking-Governance Guidance Tool (HP-GGT)**

HP-GGT is a Generic Tool

Key Informants Information Date:

KI number:

Gender:

KI works in:

- Government (Public Sector)

- Private Sector

- Non-governmental organization

- International organization

- Academia

- Media

- Other (please specify):

Agreed for second interview: Yes No

**SECTION A: Evidence-Based Questions**

**I: PARTICIPATION at policymaking level**

Everyone should have a voice in decision-making for health, either directly or through legitimate intermediate institutions that represent their interests. Such broad participation is built on freedom of association and speech, as well as capacities to participate constructively. It is about empowering citizens to have a role in decisions that affect their lives, and reach common goals by participating in policymaking process. It is the responsibility of the government to create mechanisms and spaces for participation.

**Y: Yes, N: No, P: In Process, D.K.: Don’t Know, N.A: Not Applicable**

|  | **Participation**  **Evidence Based Questions; *Answers for these questions will be collected through face-to-face interviews with KIs***  ***and answers to be validated by documented evidence*** |  |  |  |  |  |
| --- | --- | --- | --- | --- | --- | --- |
| **I. A.1** | Is there a Legal basis/requirement (Law/Regulation/Policy) to include various stakeholders in health policymaking process?  *(Assessor: Read the response options and Circle the answer given by the Key Informant)* | Y | N | P | DK | NA |
|  | **If Yes,** Specify what is it? |  |  |  |  |  |
|  | & in what phase of the policymaking process is it specified to consult with stakeholders  *(Assessor: Read the response options, allow Key Informant to reply, and check by adding (√)all options that Key Informants identifies)* |  |  |  |  |  |
|  | In policy formulation |  |  |  |  |  |
|  | In policy implementation |  |  |  |  |  |
|  | Other, Specify: |  |  |  |  |  |
|  | Not Specified |  |  |  |  |  |
|  | **If No**, is there still a commitment from the MoH/Health Authority/National Program to ensure some degree of stakeholders participation in formulation & implementation of national health policies?  *(Assessor: Read the response options and Circle the answer given by the Key Informant)* | Y | N | P | DK | NA |
| **I.A.2** | Was there a body or mechanism(s) used to involve stakeholders in policymaking process that was concerned with the development of the **X Policy**? *(Assessor: Read the response options and Circle the answer given by the Key Informant)* | Y | N | P | DK | NA |
|  | **If Yes,** what body or mechanism (s) was used to involve stakeholders in the policymaking process that was concerned with the **X Policy**?  *(Assessor: Read each response option, allow Key Informant to reply, and check by adding (√)all options that Key Informants identifies)* |  |  |  |  |  |
|  | A national committee |  |  |  |  |  |
|  | An advisory Board |  |  |  |  |  |
|  | Working Group (s) |  |  |  |  |  |
|  | Other, Specify: |  |  |  |  |  |
|  | How was this body /mechanism (mentioned above) formulated?  *(Assessor: Read each response option, allow Key Informant to reply, and check by adding (√)all options that Key Informants identifies)* |  |  |  |  |  |
|  | Formally (in written format), Specify How & By Whom: |  |  |  |  |  |
|  | Informally, Specify How: |  |  |  |  |  |
|  | If it was Formally formulated, |  |  |  |  |  |
|  | Was there a written scope/mandate for stakeholders’ involvement in the formulation of the **X Policy**?  What is the scope/mandate for the stakeholders? | Y | N | P | DK | NA |
|  | Were the roles and the responsibilities of participants for the various stakeholders specified? | Y | N | P | DK | NA |
|  | Were the qualifications of participants for the various stakeholders specified? | Y | N | P | DK | NA |
|  | Was there a timetable for the work to be carried out? | Y | N | P | DK | NA |
| **I.A.3** | Were the following stakeholders represented in the FORMULATION that was concerned with **X Policy?**  *(Assessor: Read each response option, allow Key Informant to reply, and check by adding (√)all options that Key Informants identifies)* |  |  |  |  |  |
|  | State Actors (Government, other than MoH, National, Local): Specify: |  |  |  |  |  |
|  | Health Service providers (Professional Association/Unions/Orders & Health Service Organizations/Hospital boards) Specify: |  |  |  |  |  |
|  | Parliamentary members |  |  |  |  |  |
|  | Beneficiaries (patients associations) &/or Public: Specify: |  |  |  |  |  |
|  | Civil Society: Specify: |  |  |  |  |  |
|  | Development Partners/International organizations: Specify: |  |  |  |  |  |
|  | Funders/Donors: Specify |  |  |  |  |  |
|  | Academic Institutions/Researchers: Specify: |  |  |  |  |  |
|  | Private Sector (medical, pharmaceutical industry, insurance companies): Specify: |  |  |  |  |  |
|  | Most Vulnerable or Key affected populations: Specify: |  |  |  |  |  |
|  | Media |  |  |  |  |  |
|  | Others: Specify: |  |  |  |  |  |
|  | Were representatives from local/regions within X Country represented?  How? | Y | N | P | DK | NA |
| **I.A.4** | For each category of stakeholders identified above, how were the participants involved in formulation of **X Policy** selected?  *(Assessor: Read each response option, allow Key Informant to reply, and check by adding (√)all options that Key Informants identifies)* |  |  |  |  |  |
|  | Appointed, Nominated was there a set criteria for the selection? |  |  |  |  |  |
|  | Elected, by whom? |  |  |  |  |  |
|  | Self-selected |  |  |  |  |  |
|  | Others: |  |  |  |  |  |
|  | Was their participation: |  |  |  |  |  |
|  | Voluntary |  |  |  |  |  |
|  | Mandatory |  |  |  |  |  |
|  | Are participants: *(Assessor: Read each response option, allow Key Informant to reply, and check by adding (√)all options that Key Informants identifies)* |  |  |  |  |  |
|  | Representing Themselves: Specify: |  |  |  |  |  |
|  | Representing their organizations: Specify: |  |  |  |  |  |
|  | Other, Specify: |  |  |  |  |  |
| **I.A.5** | 1. Is there a gender balance /consideration (Male vs. Female) among the stakeholders participating in the formulation of the **X Policy?** *(Assessor: Read the response options and Circle the answer given by the Key Informant)* | Y | N | P | DK | NA |
| **I.A.6** | Are there dedicated resources made available by the MoH/Health Authority to enable and facilitate participation during the policy development process of **X Policy**?  *(Assessor: Read the response options and Circle the answer given by the Key Informant)* | Y | N | P | DK | NA |
|  | **If Yes,** what type of resources is made available?  *(Assessor: Read each response option, allow Key Informant to reply, and check by adding (√)all options that Key Informants identifies)* |  |  |  |  |  |
|  | Cost of meetings (venues, coffee breaks, etc) |  |  |  |  |  |
|  | Cost of Administrative work (print outs, etc) |  |  |  |  |  |
|  | Incentives for participants (Fee or Honoraria): Specify: |  |  |  |  |  |
|  | Transportation, lodging and/or meals (Direct Payment or Reimbursement): Specify: |  |  |  |  |  |
|  | Other, Specify: |  |  |  |  |  |
| **I.A.7** | Is there documentation (Minutes of meetings) on the recommendations submitted for final decisions in relation to the formulation of the **X Policy**? *(Assessor: Read the response options and Circle the answer given by the Key Informant)* | Y | N | P | DK | NA |
|  | Are the minutes published/made available to the public?  *(Assessor: Read the response options and Circle the answer given by the Key Informant)* | Y | N | P | DK | NA |
| **I.A.8** | How final decisions were taken by participants:  *(Assessor: Read each response option, allow Key Informant to reply, and check by adding (√)all options that Key Informants identifies)* |  |  |  |  |  |
|  | Majority Vote |  |  |  |  |  |
|  | Consensus |  |  |  |  |  |
|  | Dissenting Opinions |  |  |  |  |  |
|  | Other Procedures |  |  |  |  |  |
|  | Not Specified |  |  |  |  |  |
|  | Is there documentation of this? | Y | N | P | DK | NA |
| **I.A.9** | Are the roles and responsibilities of the various stakeholders in the implementation process specified in the formulation document of the **X Policy**? *(Assessor: Read the response options and Circle the answer given by the Key Informant)* | Y | N | P | DK | NA |
|  | **If NO**, are they defined by law or by any other formal means? | Y | N | P | DK | NA |
| **I.A.10** | Is there a participatory body to oversee the implementation of the **X Policy**?  *(Assessor: Read the response options and Circle the answer given by the Key Informant)* | Y | N | P | DK | NA |
|  | **If Yes,** What is its composition? |  |  |  |  |  |
| **I.A.11** | Are other mechanism/strategies used by MOH/Health Authority/National Program to ENCOURAGE participation (express opinions/preference and encourage feedback) of different stakeholders in priority setting and in policymaking process of **X Policy**? *(Assessor: Read the response options and Circle the answer given by the Key Informant)* | Y | N | P | DK | NA |
|  | **If YES,** which mechanisms are used *(Assessor: Read each response option, allow Key Informant to reply, and check by adding (√)all options that Key Informants identifies)* |  |  |  |  |  |
|  | Opinion Polls/Surveys |  |  |  |  |  |
|  | Focus groups |  |  |  |  |  |
|  | Public Hearings/Public Comments/Citizens Juries |  |  |  |  |  |
|  | Online platforms |  |  |  |  |  |
|  | Voting |  |  |  |  |  |
|  | Hotline |  |  |  |  |  |
|  | Inter-governmental conferences |  |  |  |  |  |
|  | Policy dialogues |  |  |  |  |  |
|  | Others, specify: |  |  |  |  |  |

**II. ACCOUNTABILITY at policymaking Level**

Is about assuring that those who are responsible for designing and implementing policies are held accountable for their performance. Decision-makers in government, the private sector and civil society organizations involved in health are accountable to the public, as well as to institutional stakeholders. This accountability differs depending on the organization and whether the decision is internal or external to an organization. It is about having the right checks and balances put into place. It is ensuring that all health system actors are held publicly accountable .

**Y: Yes, N: No, P: In Process, D.K.: Don’t Know, N.A.: Not Applicable**

|  | **Accountability**  **Evidence Based Questions; *Answers for these questions will be collected through face-to-face interviews with KIs***  ***and answers to be validated by documented evidence*** | Y | N | P | DK | NA |
| --- | --- | --- | --- | --- | --- | --- |
| **II.A.1** | Does the MoH/Health Authority/National Program require signature of contract/Memorandum of understanding/Terms of Reference (that include incentives, sanctions, timeline, deliverables, etc) with stakeholders before engaging them in:  *(Assessor: Read the response options, allow Key Informant to reply, and check by adding (√)all options that Key Informants identifies)* |  |  |  |  |  |
|  | Policy Formulation |  |  |  |  |  |
|  | Policy Implementation |  |  |  |  |  |
| **II.A.2** | Are the different stakeholders (public officials & non-state actors) involved in the policy formulation related to **X Policy** formally held accountable for their contribution for decisions and policies in case of false/bad advice/failure to engage in appropriate action?  *(Assessor: Read the response options, allow Key Informant to reply, and check by adding (√)all options that Key Informants identifies)* |  |  |  |  |  |
|  | To their institutions/organizations |  |  |  |  |  |
|  | To the public |  |  |  |  |  |
|  | **If Yes,** who are the stakeholders held accountable from within institutions/organizations they represent in policy formulation?  *(Assessor: Read the response options, allow Key Informant to reply, and check by adding (√)all options that Key Informants identifies)* |  |  |  |  |  |
|  | Governmental Staff |  |  |  |  |  |
|  | Professionals |  |  |  |  |  |
|  | Private Sector |  |  |  |  |  |
|  | NGO Representatives |  |  |  |  |  |
|  | All |  |  |  |  |  |
|  | Others: Specify |  |  |  |  |  |
| **II.A.3** | What is the type of the accountability mechanisms/types used by MoH/Health Authority/National Program and various institutions/organizations involved in policy towards their representatives? *  *(Assessor: Read the response options, allow Key Informant to reply, and check by adding (√)all options that Key Informants identifies)* |  |  |  |  |  |
|  | Ethical |  |  |  |  |  |
|  | Professional/Performance |  |  |  |  |  |
|  | Legal |  |  |  |  |  |
|  | Financial |  |  |  |  |  |
|  | Others, Specify: |  |  |  |  |  |
| **II.A.4** | Does the MoH/Health Authority/National Program conduct any of the following as part of holding its staff accountable for their role in implementing the **X Policy**?  *(Assessor: Read the response options, allow Key Informant to reply, and check by adding (√)all options that Key Informants identifies)* |  |  |  |  |  |
|  | Evaluation of the performance of the individual staff on annual basis |  |  |  |  |  |
|  | Administrative/Performance audit of the relevant department(s) on annual basis |  |  |  |  |  |
|  | Financial auditing for personnel, operations, supplies, and others |  |  |  |  |  |
|  | Contracts oversight |  |  |  |  |  |
|  | Are the results of the above made public? | Y | N | P | DK | NA |
| **II.A.5** | Are any of the following tools used by MoH/Health Authority to foster accountability?**  *(Assessor: Read the response options, allow Key Informant to reply, and check by adding (√)all options that Key Informants identifies)* |  |  |  |  |  |
|  | Information System that generate key performance indicators |  |  |  |  |  |
|  | Dissemination of information |  |  |  |  |  |
|  | Participation of Public/Civil organizations |  |  |  |  |  |
|  | Whistle blowing mechanisms |  |  |  |  |  |
|  | Watchdog organizations collaboration & Protection |  |  |  |  |  |
|  | Performance incentives for good performance |  |  |  |  |  |
|  | Enforcement of rules & regulations |  |  |  |  |  |
|  | Others, Specify: |  |  |  |  |  |
| **II.A.6** | Is there monitoring & evaluation (M&E) of implementation of **X Policy?**  *(Assessor: Read the response options and Circle the answer given by the Key Informant)* | Y | N | P | DK | NA |
|  | If Yes, does the M&E include the following:  *(Assessor: Read the response options, allow Key Informant to reply, and check by adding (√)all options that Key Informants identifies)* |  |  |  |  |  |
|  | Compliance with **X Policy** by professionals/Private sector |  |  |  |  |  |
|  | Policy outcomes in terms of health improvement, efficacy, equity and quality |  |  |  |  |  |
|  | None |  |  |  |  |  |
|  | Are the results made public? | Y | N | P | DK | NA |
| **II.A.7** | **If Question II.A.6 is YES,** Is the M&E process formal?  *(Assessor: Read the response options and Circle the answer given by the Key Informant)* | Y | N | P | DK | NA |
|  | **If Yes,** |  |  |  |  |  |
|  | Is the M&E conducted independently, by whom: | Y | N | P | DK | NA |
|  | How often M&E take place? |  |  |  |  |  |
| **II.A.8** | Did the MoH/Health Authority/National Program set a formal mechanism (s) to hold implementers and/or implementing bodies from the private sector/non-state actors responsible for implementation of various components of **X Policy** accountable in-line with set timelines and targets?  *(Assessor: Read the response options and Circle the answer given by the Key Informant)* | Y | N | P | DK | NA |
|  | **If Yes,** Are the following components set? ?  *(Assessor: Read the response options, allow Key Informant to reply, and check by adding (√)all options that Key Informants identifies)* |  |  |  |  |  |
|  | Standards (accreditation, benchmarking, rules and procedures, guidelines, etc) , Specify: |  |  |  |  |  |
|  | Investigation & Answerability/Justifications Mechanisms |  |  |  |  |  |
|  | Sanctions |  |  |  |  |  |
|  | Enforcement Mechanisms |  |  |  |  |  |
|  | Rewards for Performance |  |  |  |  |  |
|  | Independent Appeal Mechanisms |  |  |  |  |  |
| **II.A.9** | **If Question II.A.8 is Yes,** Who is authority responsible for holding implementers and/or implementing body accountable?  *(Assessor: Read the response options, allow Key Informant to reply, and check by adding (√)all options that Key Informants identifies)* |  |  |  |  |  |
|  | Internal within the health sector, Specify: |  |  |  |  |  |
|  | External by independent bodies, Specify |  |  |  |  |  |
|  | External by public: Specify: |  |  |  |  |  |
|  | Others: Specify: |  |  |  |  |  |
|  | Are the results of the above made public? | Y | N | P | DK | NA |
| **II.A.10** | What are the types of sanctions applied/might be applied to implementers and/or implementing bodies responsible for implementation of **X Policy** in case of violation/not adhering to standards set? Failure to implement?  *(Assessor: Read the response options, allow Key Informant to reply, and check by adding (√)all options that Key Informants identifies)* |  |  |  |  |  |
|  | Legal Sanctions |  |  |  |  |  |
|  | Regulatory/Administrative Sanctions |  |  |  |  |  |
|  | Using Media: Name & Shame |  |  |  |  |  |
|  | Softer Sanctions, Specify: |  |  |  |  |  |
| **II.A.11** | Are there any law*(Assessor: Read the response options and Circle the answer given by the Key Informant)*s in place related to the **X Policy**? What does the law(s) cover? | Y | N | P | DK | NA |
|  | **If Yes,** is it enforced? How?  *(Assessor: Read the response options and Circle the answer given by the Key Informant)* | Y | N | P | DK | NA |
|  | Is there a plan to develop a new law? Why?  *(Assessor: Read the response options and Circle the answer given by the Key Informant)* | Y | N | P | DK | NA |

* If the answer to this question is Not Applicable, you can formulate the question as follows: What is the most suitable accountability mechanism/type that can be applied to hold the various stakeholders accountable in their role in the context of your country?

** If the answer to this question is Not applicable, you can formulate the question as follows: What of the following tools is best to be used to foster/encourage accountability in the context of your country?

**III. TRANSPARENCY at the Policymaking Level**

It is actively disclosing information on how decisions are made, implemented and evaluated. It is built on the free flow of information for all health matters. Processes, institutions, and information should be directly accessible to those concerned with them, and enough information is provided to understand and monitor health.

**Y: Yes, N: No, P: In Process, D.K.: Don’t Know, N.A.: Not Applicable**

|  | **Transparency**  **Evidence Based Questions; *Answers for these questions will be collected through face-to-face interviews with KIs***  ***and answers to be validated by documented evidence*** |  |  |  |  |  |
| --- | --- | --- | --- | --- | --- | --- |
| **III.A.1** | Is there a law/mechanism about "access to information" that allow access by the general public to government information and documents?  *(Assessor: Read the response options and Circle the answer given by the Key Informant)* | Y | N | P | DK | NA |
|  | **If Yes,** Does the law allow:  *(Assessor: Read the response options, allow Key Informant to reply, and check by adding (√)all options that Key Informants identifies)* |  |  |  |  |  |
|  | Full Access |  |  |  |  |  |
|  | Partial Access/Restricted Access |  |  |  |  |  |
|  | Access to Information on Health |  |  |  |  |  |
| **III.A.2** | Is there a law/government policy in place to promote "cyber transparency”(availability of information online) electronic government services to improve public access to government information and services?  *(Assessor: Read the response options and Circle the answer given by the Key Informant)* | Y | N | P | DK | NA |
| **III.A.3** | Is there an official website for the MoH/Health Authority?  *(Assessor: Read the response options and Circle the answer given by the Key Informant)* | Y | N | P | DK | NA |
|  | **If Yes,** |  |  |  |  |  |
|  | Is it user-friendly? | Y | N | P | DK | NA |
|  | Is it updated on regular basis/has up to date news, documents, etc? | Y | N | P | DK | NA |
|  | Is access to the website open to all? | Y | N | P | DK | NA |
|  | **If No,** Why? who is allowed to access it? |  |  |  |  |  |
| **III.A.4** | Are decisions related to priority setting in relation to the **X Policy** made public?  *(Assessor: Read the response options and Circle the answer given by the Key Informant)* | Y | N | P | DK | NA |
|  | How? |  |  |  |  |  |
| **III.A.5** | Are decisions related to resource allocation (general resource allocation decisions, focused on overall budgets) in relation to the **X Policy** made public?  *(Assessor: Read the response options and Circle the answer given by the Key Informant)* | Y | N | P | DK | NA |
|  | How? |  |  |  |  |  |
|  | Are the Cost estimates clearly explained and justified? | Y | N | P | DK | NA |
| **III.A.6** | Is there official, up-to-date (within last 5 years), detailed policy document regarding **X Policy**?  *(Assessor: Read the response options and Circle the answer given by the Key Informant)* | Y | N | P | DK | NA |
|  | **If Yes,** Is it: |  |  |  |  |  |
|  | Publicly available? | Y | N | P | DK | NA |
|  | Easily accessible? | Y | N | P | DK | NA |
|  | Available on the MoH/Health authority website? | Y | N | P | DK | NA |
|  | Is the document available in the official/national language of the country?  What other languages is it available? | Y | N | P | DK | NA |
| **III.A.7** | Does the document related to **X Policy** include the following information:  *(Assessor: Read the response options, allow Key Informant to reply, and check by adding (√)all options that Key Informants identifies)* |  |  |  |  |  |
|  | Background on how the policy was formulated (based on international guidelines, best practices, etc) |  |  |  |  |  |
|  | Objectives, Purpose and goals based on priority problems |  |  |  |  |  |
|  | Evidence used to inform policy formulation |  |  |  |  |  |
|  | Mechanisms to engage stakeholders participation |  |  |  |  |  |
|  | Stakeholders (Names & Affiliation) who participated/consulted in policy formulation |  |  |  |  |  |
|  | How decisions were made/Justifications for decisions |  |  |  |  |  |
|  | Other factors that influenced the policy formulation: Specify: |  |  |  |  |  |
|  | Responsible body for releasing or approving the policy |  |  |  |  |  |
|  | Contracting requirements for implementation if needed |  |  |  |  |  |
|  | Time frame for implementation |  |  |  |  |  |
|  | Measurable Indicators & Targets |  |  |  |  |  |
|  | Plans for monitoring & evaluation |  |  |  |  |  |
|  | Funding requirements/allocation (including costs of human resources, medicines, management, infrastructure and costs for activities and stakeholders beyond the public health sector) |  |  |  |  |  |
|  | Intended audience of the document |  |  |  |  |  |
| **III.A.8** | Is there plans to publish/already published any of the following documents that are related to implementation of **X Policy**?  *(Assessor: Read the response options, allow Key Informant to reply, and check by adding (√)all options that Key Informants identifies)* |  |  |  |  |  |
|  | Five year strategic plan/Operational Plan/Implementation Plans |  |  |  |  |  |
|  | Program/Project Documents |  |  |  |  |  |
|  | Relevant MoH/Health Authority decisions |  |  |  |  |  |
|  | Policy Evaluation Reports |  |  |  |  |  |
|  | Financial reports including how funds were generated/secured for implementation/source of funding |  |  |  |  |  |
|  | Scientific Publications |  |  |  |  |  |
|  | Contracts made for implementation |  |  |  |  |  |
|  | Details about recruitment made to implement |  |  |  |  |  |
|  | Others: Specify |  |  |  |  |  |
| **III.A.9** | 1. Does the MoH/Health Authorities/National Program release information related to formulated and implemented X policy in "predictable manner"/Periodic/regular manner? 2. *(Assessor: Read the response options and Circle the answer given by the Key Informant)* | Y | N | P | DK | NA |
| **III.A.10** | Did participants declared any conflict of interest by signing an official form?  *(Assessor: Read the response options and Circle the answer given by the Key Informant)* |  |  |  |  |  |
|  | In the policy formulation | Y | N | P | DK | NA |
|  | In the policy implementation | Y | N | P | DK | NA |
|  | **If Yes,** Is there a policy on conflict of interest management? | Y | N | P | DK | NA |
|  | Who is responsible for the oversight on conflict of Interest? |  |  |  |  |  |
| **III.A.11** | Which of the following methods, if any, the MoH/Health Authority/National Program is using or has used in the past 12 months, to INFORM/Disseminate to stakeholders (including the public) about policy formulation, development, implementation and progress of **X Policy**?  *(Assessor: Read the response options, allow Key Informant to reply, and check by adding (√)all options that Key Informants identifies)* |  |  |  |  |  |
|  | Use of Mass Media (TVs, Radios, etc) |  |  |  |  |  |
|  | Wide Advertisement (Newspapers, Billboards, etc) |  |  |  |  |  |
|  | Bulletins/Newsletters |  |  |  |  |  |
|  | Targeted Personal Invitations by email, mail, Telephone, etc.. |  |  |  |  |  |
|  | Social Media |  |  |  |  |  |
|  | Smart Phones Applications |  |  |  |  |  |
|  | Others, specify: |  |  |  |  |  |

**IV. USE OF INFORMATION at the policymaking Level**

Is essential for a good understanding of health system without which it is not possible to provide evidence for informed decisions that influences the behavior of different interest groups that support, or at least do not conflict with, the strategic vision for health. It includes; information generation, collection, analysis and dissemination. Sound and reliable information is essential for health system policy development and implementation, governance and regulation. Availability of information includes accessibility, user-friendly, comprehensiveness and completeness.

**Y: Yes, N: No, P: In Process, D.K.: Don’t Know, N.A.: Not Applicable**

|  | **Information**  **Evidence Based Questions; *Answers for these questions will be collected through face-to-face interviews with KIs***  ***and answers to be validated by documented evidence*** |  |  |  |  |  |
| --- | --- | --- | --- | --- | --- | --- |
| **IV.A.1** | Is the MoH/Health Authority/National Program directly involved in the following in relation to policymaking:  *(Assessor: Read the response options, allow Key Informant to reply, and check by adding (√)all options that Key Informants identifies)* |  |  |  |  |  |
|  | Information Generation |  |  |  |  |  |
|  | Dissemination of health information, Specify type of information disseminated: |  |  |  |  |  |
|  | Publication, Specify types of publications: |  |  |  |  |  |
|  | Knowledge Translation to policy |  |  |  |  |  |
| **IV.A.2** | Is the MoH/Health Authority/National Program using any of the following?  *(Assessor: Read the response options, allow Key Informant to reply, and check by adding (√)all options that Key Informants identifies)* |  |  |  |  |  |
|  | Data Collection tools (vital registries, surveys (population, facilities, etc), health statistics), Specify: |  |  |  |  |  |
|  | Data Management technologies, Specify: |  |  |  |  |  |
|  | Validation of Data sources |  |  |  |  |  |
|  | Checking sources of funding of research to be used in policy |  |  |  |  |  |
| **IV.A.3** | Does the MoH/Health Authority/National Program have any form of partnership/collaboration with research centers inside as well as outside the country?  *(Assessor: Read the response options and Circle the answer given by the Key Informant)* | Y | N | P | DK | NA |
|  | **If Yes,** Does the MoH/Health Authority/National Program allocate funds in its yearly budget for research related to policy? | Y | N | P | DK | NA |
| **IV.A.4** | Does MoH/Health Authorities make **Raw** data generated at health facilities/health service delivery level accessible to researchers?  *(Assessor: Read the response options and Circle the answer given by the Key Informant)* | Y | N | P | DK | NA |
| **IV.A.5** | Is there a specialized unit/staff in the MoH/Health Authority/National Program that deals with research analysis for policymaking?  *(Assessor: Read the response options and Circle the answer given by the Key Informant)* | Y | N | P | DK | NA |
| **IV.A.6** | Was the developed **X Policy** informed by scientific evidence?  *(Assessor: Read the response options and Circle the answer given by the Key Informant)* | Y | N | P | DK | NA |
| **IV.A.7** | **If Questions IV.A.6 is YES,** Which of the following criteria were used for the inclusion of scientific evidence in policy formulation of the **X Policy?**  *(Assessor: Read the response options, allow Key Informant to reply, and check by adding (√)all options that Key Informants identifies)* |  |  |  |  |  |
|  | Reliable and of good quality source/Peer reviewed studies |  |  |  |  |  |
|  | Up-to-date (published in the last 5 years) |  |  |  |  |  |
|  | Comprehensive/Extensive |  |  |  |  |  |
|  | Locally Appropriate |  |  |  |  |  |
|  | Easily Accessible |  |  |  |  |  |
|  | Global/International |  |  |  |  |  |
|  | National |  |  |  |  |  |
|  | Local Evidence/Community level |  |  |  |  |  |
|  | Only Available evidence |  |  |  |  |  |
| **IV.A.8** | Were other types of information utilized in the policy formulation of **X Policy**, Like:  *(Assessor: Read the response options, allow Key Informant to reply, and check by adding (√)all options that Key Informants identifies)* |  |  |  |  |  |
|  | Experts opinion |  |  |  |  |  |
|  | Financial information |  |  |  |  |  |
|  | Governing laws |  |  |  |  |  |
|  | Political direction & commitment |  |  |  |  |  |
|  | Others, Specify: |  |  |  |  |  |
| **IV.A.9** | Does the MoH/Health Authority/National Program publish/plan to publish periodic progress reports/M&E reports on policy implementation status of **X Policy**? | Y | N | P | DK | NA |
|  | **If Yes,** Does progress reports include:  *(Assessor: Read the response options, allow Key Informant to reply, and check by adding (√)all options that Key Informants identifies)* |  |  |  |  |  |
|  | Follow Up Plans |  |  |  |  |  |
|  | Impact of the policy |  |  |  |  |  |
|  | Recommended review of the policy considering results obtained |  |  |  |  |  |
|  | **If Yes,** Are the progress reports disseminated? | Y | N | P | DK | NA |
|  | **If Yes,** is it disseminated to |  |  |  |  |  |
|  | Public |  |  |  |  |  |
|  | Only for stakeholders |  |  |  |  |  |
|  | **What media/means are used to disseminate the results?** |  |  |  |  |  |
|  | Printed material |  |  |  |  |  |
|  | Public Presentations |  |  |  |  |  |
|  | Website |  |  |  |  |  |
|  | - Others, Specify |  |  |  |  |  |
| **IV.A.10** | **If Questions IV.A.10 is YES,** What are the objectives of progress reports?  *(Assessor: Read the response options, allow Key Informant to reply, and check by adding (√)all options that Key Informants identifies)* |  |  |  |  |  |
|  | Increase awareness |  |  |  |  |  |
|  | Evaluate the situation |  |  |  |  |  |
|  | Identify problems |  |  |  |  |  |
|  | Provide information |  |  |  |  |  |
|  | Assign responsibility |  |  |  |  |  |

**V. RESPONSIVENESS TO POPULATION NEEDS at the policy making level**

Institutions and processes should try to serve all stakeholders to ensure that the policies and programs are responsive to the health and non-health needs of its users. Governments are obliged to listen to the needs of their citizens and act on their concerns, and respond to their expectations. It is not only about "Clinical" Responsiveness.

**Y: Yes, N: No, P: In Process, D.K.: Don’t Know, N.A.: Not Applicable**

|  | **Responsiveness**  **Evidence Based Questions; *Answers for these questions will be collected through face-to-face interviews with KIs***  ***and answers to be validated by documented evidence*** | | Y | N | P | DK | NA |
| --- | --- | --- | --- | --- | --- | --- | --- |
| **V.A.1** | Does the **X Policy** include an objective/goal that MoH/Health Authority/National Program will ensure access to adequate **Quality** of care services to ALL the population/patients including disadvantaged/vulnerable groups to be covered by the policy? *(Assessor: Read the response options and Circle the answer given by the Key Informant)* | Y | | N | P | DK | NA |
| **V.A.2** | Does the **X Policy** include an objective/goal that the health services will respect the confidentiality and the dignity of the population/patients?  *(Assessor: Read the response options and Circle the answer given by the Key Informant)* | Y | | N | P | DK | NA |
| **V.A.3** | Does the **X Policy** include an objective/goal that the health providers will respect the rights of the patients in terms of:  *(Assessor: Read the response options, allow Key Informant to reply, and check by adding (√)all options that Key Informants identifies)* |  | |  |  |  |  |
|  | Autonomy to participate in health related decisions |  | |  |  |  |  |
|  | Freedom of choice of health care provider |  | |  |  |  |  |
|  | Provide all information related to the patients’ medical conditions in an understandable manner |  | |  |  |  |  |
|  | Others: Specify: |  | |  |  |  |  |
| **V.A.4** | Does the **X Policy** refer to the explicit package of benefits to be provided to patients at the different levels of care?  *(Assessor: Read the response options and Circle the answer given by the Key Informant)* | Y | | N | P | DK | NA |
| **V.A.5** | Does the **X Policy** include an objective/goal that the health services will be provided to population/patients within reasonable timeframe?  *(Assessor: Read the response options and Circle the answer given by the Key Informant)* | Y | | N | P | DK | NA |
|  | **If Yes,** is the timeframe specified? | Y | | N | P | DK | NA |
| **V.A.6** | Does the **X Policy** refer to how referral of patients will take place from one level of care to the other?  *(Assessor: Read the response options and Circle the answer given by the Key Informant)* | Y | | N | P | DK | NA |
|  | **If Yes,** is the timeframe for referral specified? |  | |  |  |  |  |
| **V.A.7** | Does the **X Policy** include an objective/goal to set in place an official complaint mechanism?  *(Assessor: Read the response options and Circle the answer given by the Key Informant)* | Y | | N | P | DK | NA |
| **V.A.8** | Was a needs assessment (targeting the public)/Public Opinion surveys conducted as part of the **X Policy** formulation process?  *(Assessor: Read the response options and Circle the answer given by the Key Informant)* | Y | | N | P | DK | NA |
|  | **If Yes,** Is there evidence that the identified population needs were incorporated in the **X Policy**? | Y | | N | P | DK | NA |
| **V.A.9** | Do the monitoring & evaluation plans of the **X Policy** include a component to assess whether the policy is meeting the population needs through conducting patients satisfaction surveys/exit surveys?  *(Assessor: Read the response options and Circle the answer given by the Key Informant)* | Y | | N | P | DK | NA |
|  | **If Yes,** is it recommended to be done on regular basis? | Y | | N | P | DK | NA |
| **V.A.10** | Did the MoH/Health Authority/National Program develop a communication strategy to inform the public about the X Policy? *(Assessor: Read the response options and Circle the answer given by the Key Informant)* | Y | | N | P | DK | NA |

**SECTION B: Perception-based Questions**

1. **Participation at the policymaking level**

|  | **Participation**  **KI Interviews Questions; *These questions will be asked to key informants through face-to-face In-depth interviews*** |
| --- | --- |
| **I.B.1** | How do you view the role of MoH/Health Authorities/National Program in encouraging stakeholders’ participation in policy formulation and implementation in general? & in the **X Policy** development in specific? Does the MoH/Health Authority/National Program has the institutional capacity and needed resources to facilitate the participation process? In terms of leadership? Planning? Needed information? Institutional arrangements? Database of key stakeholders? |
| **I.B.2** | To what extent was the formulation process of the **X Policy** inclusive of the key stakeholders? Were they “Effectively “consulted**?** Were all relevant voices taken into account? Which stakeholders were missing? |
| **I.B.3** | What type of process was applied for the selection/identification of participants in the **X Policy** formulation? Do You consider that it was a fair/effective process to ensure a qualified group? A representative group? Why? |
| **I.B.4** | Who were the powerful stakeholders in the decision making/formulation of the **X Policy**? Was their influence hindering or facilitating the formulation process of **X Policy**? What their influence led to? |
| **I.B.5** | What are the barriers and/or facilitators to the participatory process? For MoH/Health Authorities/National program? For stakeholders? |
| **I.B.6** | What are the mechanisms used to enable stakeholder participation in policymaking process? Do they include mechanisms to give voice to the traditionally voiceless groups (homeless, migrants/refugees, unemployed, minorities, disabled, elderly, etc?? How do you view the effectiveness of these mechanisms? |

**II. Accountability at the policymaking level**

|  | **Accountability**  **KI Interviews Questions; *These questions will be asked to key informants through face-to-face in-depth interviews*** |
| --- | --- |
| **II.B.1** | To what extent do you agree that all stakeholders should be held accountable for their role in the policymaking process including formulation?  What is the best way to hold the various stakeholders accountable for their role in policymaking? How to ensure that they know they will be held accountable prior to their involvement? |
| **II.B.2** | What is the role of media in accountability in policymaking in your setting? Is media playing a positive or negative role in **Policy X**? Give examples |
| **II.B.3** | Does the civil society have an active role as watchdogs over policy formulation and implementation of **Policy X**? How? Give examples |
| **II.B.4** | How the public can hold various stakeholders accountable for their role in policymaking in general and in relation to **Policy X**? |
| **II.B.5** | How the implementing bodies are held accountable for their roles in the policy implementation process of **X Policy**? Are all held accountable in equal manner? Give examples |
| **II.B.6** | How is the law (s) related to **X Policy** translated into rules, regulations and procedures? Who is responsible for this? How does the MoH/Health Authority/National Program ensure that regulations, legislations and sanctions are fairly enforced in relation to the implementation of the **X Policy** in both public and private sector? |

**III. Transparency at the Policymaking level**

|  | **Transparency**  **KI Interviews Questions; *These questions will be asked to key informants through face-to-face in-depth interviews*** |
| --- | --- |
| **III.B.1** | Does the MoH/Health authority/National Program have the interest/willingness/Commitment to achieve better transparency? What is the type of this willingness/commitment? How can the MoH/Health Authority/National Program increase its transparency in the policymaking process? Does the MoH/Health Authority/National Program have the needed capacity/means to achieve better transparency? |
| **III.B.2** | 1. How the MoH/Health Authorities/National Program can ensure that the opinions of the different stakeholders are documented & disclosed/published as part of a transparent policymaking process? |
| **III.B.3** | 1. How transparent was the policy formulation process of **X Policy** as perceived by stakeholders? by public? What made it transparent? What could have been done to make it more transparent? |
| **III.B.4** | 1. How comprehensive is the **X Policy?** Is the policy document user-friendly& easily accessible? How useful? What is missing? |
| **III.B.5** | 1. How transparent was the process of priority setting during the development of **X Policy**? How this process can be improved? |
| **III.B.6** | 1. How transparent is/was the process of resource allocation for implementing the **X Policy**? Are criteria applied for allocating resources known to all? |

**IV. Information at the policymaking level**

|  | **Information**  **KI Interviews Questions; *These questions will be asked to key informants through face-to-face in-depth interviews*** |
| --- | --- |
| **IV.B.1** | How committed is the MoH/Health Authority/National Program leadership to use evidence-based (scientific evidence) and other types of information in policymaking process? What is the evidence for this commitment? Is it documented? |
| **IV.B.2** | How can the capacity of staff at the MoH/Health Authority/National Program be improved in terms of to access/use and analysis of research evidence? |
| **IV.B.3** | Can you describe the relationship between MoH/Health Authority/National Program leadership and researchers? Is their regular interaction? |
| **IV.B.4** | Do you consider the scientific evidence used in the formulation of **X Policy** pertinent/adequate? Why? What factors influenced the uptake of evidence-based/research findings into **X Policy**? What additional evidence would have been necessary? |
| **IV.B.5** | How national evidence is generated? What is the role of MoH/Health Authority/National Program in adapting research findings to local context? Give Examples in relation to the **X Policy.** |
| **IV.B.6** | What other factors (other than evidence-base political context for example, ) contributed to the formulation of X **Policy**? |

**V. Responsiveness at the policymaking level**

|  | **Responsiveness**  **KI Interviews Questions; *These questions will be asked to key informants through face-to-face in depth interviews*** |
| --- | --- |
| **V.B.1** | How do you view MoH/Health Authority/National Program institutional capacity to collect/gather public needs/preferences to be incorporated into policymaking process? What Mechanisms can be used to improve policy responsiveness to the population needs? |
| **V.B.2** | What are the factors that can positively or negatively influence the responsiveness of MoH/Health Authorities/National Program to the public needs in the policymaking process? |
| **V.B.3** | How does the MoH/Health Authority/National Program balance the competing interests/conflicting needs and influence of professionals/elite groups with public opinion (if there is any conflict present between the two opinions)? And between different groups of elites, or different publics or different social groups? |
| **V.B.4** | Does the MoH/Health Authorities/National Program usually respond to media and/or civil societies reports regarding failure to implement policies? How the response is formulated? Please Give Examples |
| **V.B.5** | How responsive is the **X Policy** to the population needs in general? And to the vulnerable population needs in particular? Is the policy patient-centered? Please explain |
| **V.B.6** | How do you perceive the timeliness as well as the promptness of developing the **X Policy** in response to population legitimate needs? |

**Exit Interview Questions:**

- If you were in High level authority, what would have you done differently?
- Please provide any additional comments if you like

**Thank you for your participation in this guidance tool.**

**Your responses will help to better guide MOHs/Health Authorities/National Program to strengthen governance of the policymaking process that they lead at the national level.**
