# Supplementary material for: Assessing the governance of the health policy-making process using a new governance tool: the case of Lebanon
Source: Health Res Policy Syst. 2020 Jun 15;18:66. doi: 10.1186/s12961-020-00557-1 (PMC7294613; doi:10.1186/s12961-020-00557-1)
Supplement: Supplementary file 2 — Additional file 2. Traffic lights summary and summary of findings by principle and characteristics; data presentation of the pilot results that was submitted to policy-makers. [file 12961_2020_557_MOESM2_ESM.docx]

Additional File 2A: Traffic Lights Summary

| **Participation** |  |
| --- | --- |
| Legal basis/requirement (law/regulation/policy) to include various stakeholders in the health policymaking process |  |
| A commitment to ensure some degree of stakeholder participation in formulation and implementation |  |
| A body or mechanism(s) employed to involve stakeholders in the development of the mental health policy, working group |  |
| Formally formulated |  |
| A written scope/mandate for stakeholder involvement in the formulation of the mental health policy exists |  |
| Roles and the responsibilities of participants for various stakeholders are specified |  |
| Qualifications of the participants for various stakeholders are specified |  |
| Timetable to perform the work |  |
| The various stakeholders represented in the formulation of mental health policy included the following: |  |
| State actors (government, other than the MoH, national, local) |  |
| Health service providers |  |
| Parliamentary members |  |
| Beneficiaries (patient associations) and/or Public |  |
| Civil society |  |
| International organisations |  |
| Funders/financiers |  |
| Academic institutions/researchers |  |
| Private sector (medical, pharmaceutical industry, insurance companies) |  |
| Most vulnerable or key affected populations |  |
| Media |  |
| Participants involved in the formulation of mental health policy were as follows: |  |
| Appointed |  |
| Elected |  |
| Representing their organisations |  |
| Gender balance/consideration (male versus female) among the stakeholders participating was considered |  |
| Dedicated resources made available to enable participation included the following: |  |
| Cost of meetings (venue, coffee breaks, and printouts) |  |
| Incentives for participants (fee or honoraria) |  |
| Transportation (direct payment or reimbursement) |  |
| Documentation (minutes of meetings) exists |  |
| Minutes published/made available to the public |  |
| Final decisions were taken by participants: |  |
| Consensus |  |
| Various stakeholders to be involved in the implementation of the mental health policy included: |  |
| State actors (government) |  |
| Health service providers |  |
| Beneficiaries (patient associations) and/or Public |  |
| Civil society |  |
| Development partners |  |
| Academic institutions/researchers |  |
| Private sector (pharmaceutical industry, insurance companies) |  |
| Media |  |
| Local authorities/community-based organisations |  |
| Roles and responsibilities of the various stakeholders in the implementation process specified |  |
| Participatory body to oversee the implementation of the mental health policy |  |
| Strategies used by the MOH/Health authority to encourage participation by different stakeholders in policymaking in mental health |  |
| Opinion polls/surveys |  |
| Focus groups |  |
| Online platforms |  |
| Voting |  |
| Hotline |  |
| Policy dialogues |  |

| **Accountability** |  |
| --- | --- |
| MoH/health authority requires signature of contracts/MoU with various stakeholders before engaging them in: |  |
| Policy formulation |  |
| Policy implementation |  |
| Formal mechanism(s) followed by MoH/health authorities to hold public officials and non-state stakeholders involved in the policy formulation accountable |  |
| Stakeholders are held accountable as: |  |
| Institutions/organisations represented |  |
| Individuals are represented |  |
| Accountability types used by MoH/health authority to hold various stakeholders accountable |  |
| Ethical |  |
| Professional/performance |  |
| Legal |  |
| Financial |  |
| MoH/health authority holds its staff accountable for implementing the mental health policy by conducting: |  |
| Evaluation of the performance of the individual staff on an annual basis |  |
| Administrative/performance audit of the relevant department(s) on an annual basis |  |
| Contracts’ oversight |  |
| Various stakeholders are aware of this process/results made public |  |
| Formal mechanism(s) to hold implementing bodies accountable in line with set timelines and targets exist |  |
| Internal within the health sector |  |
| External by independent bodies |  |
| External by the public |  |
| Various stakeholders who are aware of this process/results made public |  |
| Components of accountability mechanism(s) used by MoH/health authority at all levels are in place and include: |  |
| Set standards |  |
| Investigation and answerability/justifications |  |
| Sanctions |  |
| Enforcement |  |
| Rewards for performance |  |
| Appeals |  |
| Tools used by MoH/health authority to foster accountability include: |  |
| Information system that generates key performance indicators |  |
| Dissemination of information |  |
| Participation of public/civil organisations |  |
| Whistle blowing mechanisms |  |
| Watchdog organisations collaboration and protection |  |
| Performance incentives for good performance* |  |
| Enforcement of rules and regulations** |  |
| Appeal mechanisms |  |
| Monitoring and evaluation (M&E) of mental health policy exists and includes**:** |  |
| Compliance with mental health policy by professionals/private sector |  |
| Policy outcomes in terms of health improvement, efficacy, equity, and quality |  |
| Various stakeholders who are aware of this process/results made public |  |

| M&E process is formal |  |
| --- | --- |
| M&E conducted independently*** |  |
| Types of sanctions applied/might be applied to implementing bodies in case of violation/not adhering to standards set/ failure to implement |  |
| Legal sanctions |  |
| Regulatory/administrative sanctions |  |
| Using media: Name and shame |  |
| Softer sanctions |  |
| Laws in place related to the mental health policy |  |
| Enforced |  |
| A plan to develop a new law**** |  |

| **Transparency** |  |
| --- | --- |
| A law/mechanism that allows the general public access to government information and documents |  |
| A law/government policy in place to promote electronic government services to improve public access to government information and services* |  |
| Official website for the MoH/health authority |  |
| User-friendly |  |
| Updated on regular basis |  |
| Access to the website open to all |  |
| Decisions related to priority setting in relation to the mental health policy made public |  |
| Decisions related to resource allocation regarding the mental health policy made public |  |
| Official, up-to-date (within last five years), and detailed policy document regarding mental health policy |  |
| Publicly available |  |
| Easily accessible |  |
| Available on the MoH/health authority website |  |
| Available in the official/national language of the country |  |
| Document related to mental health policy includes the following information: |  |
| Background on how the policy was formulated (based on international guidelines, best practices, among other things) |  |
| Objectives, purpose, and goals based on priority problems |  |
| Evidence used to inform policy formulation |  |
| Mechanisms to engage stakeholder participation |  |
| Stakeholders (names and affiliation) who participated/consulted in policy formulation |  |
| How decisions were made/justifications for decisions |  |
| Other factors that influenced the policy formulation |  |
| Body responsible for releasing or approving the policy |  |
| Clear distribution of responsibility for implementation |  |
| Contracting requirements for implementation if required |  |
| Time frame for implementation |  |
| Measurable indicators and targets** |  |
| Plans for monitoring and evaluation |  |
| Funding requirements/allocation |  |
| Intended audience of the document |  |
| Official publication(s) related to implementation of mental health policy available, such as: |  |
| Five-year strategic plan/operational plan |  |
| Programme/project documents |  |
| Relevant MoH/health authority decisions |  |
| Progress reports*** |  |
| Financial reports including how funds were generated/secured for implementation/source of funding |  |
| Policy evaluation**** |  |
| Scientific publications |  |
| Contracts made for implementation |  |
| Details about recruitment made for implementing |  |
| 1. MoH/health authorities release information related to formulated and implemented policies in a periodic/regular manner***** |  |
| Participants declared any conflict of interest by signing an official form |  |
| In the policy formulation |  |
| In the policy implementation |  |
| A policy on conflict of interest management exists |  |
| MoH/health authority is using or has used in the past 12 months, to inform/disseminate to stakeholders (including the public) about mental health policy: |  |
| Use of mass media |  |
| Wide scale advertisement |  |
| Bulletins/newsletters |  |
| Targeted personal invitations |  |
| Contact by email, telephone, mail |  |
| Website |  |
| Social media |  |
| Smart phones applications |  |

*Government policy in place to promote electronic government services to improve public’s access to government information and services: It is a government policy to simplify procedures and improve access to services using electronic services, but stakeholders are not aware of this policy

**The strategy includes targets but not indicators

***Progress reports are not published and are shared with some and not all stakeholders

****Policy evaluations are not developed yet as it is still too early to do so

*****Stakeholders believe that the national programme is not publishing relevant information in a regular manner, although a newsletter is published every three months; but, this requires better dissemination

| **Information** |  |
| --- | --- |
| MoH/health authority directly involved in the following in relation to policymaking: |  |
| Information generation |  |
| Dissemination of health information |  |
| Publication |  |
| Knowledge translation to policy* |  |
| MoH using: |  |
| Data collection tools, specify: examples: vital registries, surveys (population, facilities, etc), health statistics |  |
| Data management technologies, specify: |  |
| Validation of data sources |  |
| MoH/health authority has a form of partnership/collaboration with research centres |  |
| MoH/health authority allocates funds in its yearly budget for research related to policy |  |
| MoH/health authorities make raw data generated at health facilities/health service delivery level accessible to researchers |  |
| A specialised unit/staff in the MoH/health authority to deal with research analysis for policymaking exists |  |
| MoH/health authorities have a mechanism in place to check sources of funding of research to be used in policy |  |
| Mental health policy was informed by scientific evidence |  |
| The scientific evidence used in policy formulation of the mental health policy is as follows: |  |
| Reliable and of good quality source/peer-reviewed studies |  |
| Up to date (published in the last 5 years) |  |
| Comprehensive |  |
| Locally appropriate |  |
| Easily accessible |  |
| Global |  |
| National |  |
| Other types of information utilised in the policy formulation of mental health policy |  |
| Financial information |  |
| Governing laws |  |
| Political direction and commitment |  |
| Public opinion |  |
| MoH/health authority produces periodic progress reports/M&E reports on mental health policy |  |
| Progress reports are disseminated to the public |  |
| Progress reports are disseminated only to stakeholders** |  |

| The following are used to disseminate: |  |
| --- | --- |
| Printed material; flyers |  |
| Website |  |
| Emails |  |
| Objectives of progress reports |  |
| Increase awareness |  |
| Judge the situation/identify problems |  |
| Provide evidence |  |
| Assign responsibility |  |

*Knowledge translation to policy is not used yet, but there are plans to use it

**Progress reports are not disseminated to all stakeholders

| .**Responsiveness** |  |
| --- | --- |
| Mental health policy provides for/ensures that it will give access to quality services for all the population/patients including disadvantaged/vulnerable groups to be covered by the policy. |  |
| Mental health policy provides for/ensures that the health services will respect the confidentiality and dignity of the population/patients. |  |
| Mental health policy refers to the rights as well as the responsibilities of the patients/user clearly.* |  |
| Mental health policy refers to the explicit benefit package to be provided to the patients at the different levels of care. |  |
| Mental health policy provides for/ensures that health services will be provided to patients within reasonable timeframe. |  |
| Mental Health Policy refers to how the referral of patients will take place from one level of care to the other.. |  |
| Needs assessment was conducted as part of the mental health policy formulation process |  |
| Monitoring and evaluation plans of the mental health policy include a component to assess whether the policy is meeting the population’s needs through conducting patient satisfaction surveys/exit surveys. |  |

*The strategy mentions the rights but not the responsibilities of the patients/users.

**There are plans to assess whether the policy is meeting the needs of the population; this emerged as a result of this assessment

**Additional File 2B: Summary of Findings by Principles & Characteristics**

| **Principle** | **Characteristics** | **Summary of Findings** |
| --- | --- | --- |
| **Participation**  **Participation** | - Legal framework  - Not all types of  participants were involved  - Institutional official structure  - SOPs  - Financial Resources  - Transparency  - Mechanisms used to enhance participation  - Participatory body to oversee implementation  - Barriers of participation  - Consensus orientation  - Financial Resources & contextual factors  - Leadership  - Facilitators of participation | **GAPS**  - There is **no** legal requirement to include various stakeholders in health policymaking  - **No** participation of parliamentary members, private sector and patient groups  - There was a working group but not officially established  - **No** mandate for the committee; roles, responsibilities and qualifications are not specified  - **No** incentives were given for participation but meeting costs were secured  - Minutes of meetings are documented but **not** published  - Role and the responsibilities of the implementation bodies were **not** set within the strategy  - **No** strategies were used to encourage participation like opinion polls, focus groups, online platforms, etc  - **No** participatory body was established to oversee the implementation of the strategy  - **No** coordination at the implementation  - Barriers to the process included: finding the right time and place for the meetings, was not an easy task to agree on same agenda, terminologies, etc, information given before the meeting was not enough |
|  |  | **STRENGTHS**  - Decisions were taken by consensus  - Implementation will count on external funding which will not be sustainable  - There is commitment of the national team to involve all in strategy formulation  - Facilitators of the process included: leadership of the mental health team and the way discussions were handled, all stakeholders were motivated, almost all stakeholders knows each other. |
| **Accountability**  **Accountability** | - MOUs  - Formal mechanisms for accountability  - Types of accountability  - External accountability by external  - Components of accountability  - Mechanism to foster accountability  - Performance accountability  - Types of sanctions  - Rule of law  - Mechanism to foster accountability  -Internal accountability  - Role of Media  - Role of NGOs | **GAPS**  - Stakeholders did **not** sign any MOU before their engagement in the policy formulation process  - **No** formal mechanisms in place to hold public officials and non-state stakeholders involved in the policy formulation accountable  - Most used type of accountability is ethical, while others like: legal, performance and financial are **not** enforced  - **No** external impendent audit takes place nor accountability by the public is practiced  - All components of accountability are **not** in place: standards, investigation mechanisms, sanctions, enforcement nor appeals  - There is **no** mechanisms for whistle blowing and watchdog protection  - Monitoring and evaluation (M&E) plans do **not** include evaluation for the compliance of private sector with implementing the strategy  - There is **no** set sanctions (legal, regulatory, softer sanctions) applied in case of violation or failure to implement  - Current law about mental health is **not** enforced and there are plans to amend it |
|  |  | **STRENGTHES**  - MoH is involved in generating key performance indicators and dissemination of information  - MoH has an internal process to hold its staff responsible for implementation accountable using evaluation of performance, administrative audit and contacts oversight, but result of those are not published  - Media has an important role in accountability, but they need to be sensitized and trained, and a toolkit for media is needed  - NGOs has also a big and active role to put pressure on MoH and to implement policies |
| **Transparency**  **Transparency** | -Laws  - Transparency in Priority Resource Allocation  - Documentation of policy  - COI Declaration  - Full transparency  - E-Transparency  - Transparency in Priority Setting  - Release of governments decisions  - Dissemination channels | **GAPS**  - There is **no** law in place to allow access to government information by the public  - Priorities setting process was **not** transparent, as the national team suggested the priorities and consensus followed and there were no justifications for the goals set  - Decisions related to resource allocation are **Not** made public  - The strategy document do **not** include: mechanisms used to engage the stakeholders, justifications for decisions that were made, clear distributions of responsibilities for implementation, funding requirements and intended audience of the document  - Participants did **not** declare any conflict of interest before being engaged in the consultation process of developing the strategy  - There is **no** official documents related to five year strategic plan, relevant implementation decisions, financial reports, contracts made for implementation nor details about recruitment made to implement  - Transparency can burden the process  - The process was transparent to stakeholders but **not** to the public  - Stakeholders were informed on regular basis on the development process **not** on implementation plans  - The strategy document is comprehensive enough and user friendly but it is **not** made for the public as it contains scientific terms  - Roles and responsibilities of the various stakeholders are **not** defined and not transparent, there is a lack of planning for the implementation |
|  |  | **STRENGTHS**  - There is an official website that is updated, user friendly and is accessible to all public  - Decisions related to priority setting are made public  - There is an official document about the strategy that is updated, publicly available, placed on website, and is available in the national language of the country which is Arabic  - The strategy document include the following: Background on how the policy was formulated, Objectives, Purpose and goals based on priority problems, evidence used, stakeholders who were involved, responsible body for releasing the strategy, timeframe for implementation, and plans for M&E.  - MoH disseminated information about the strategy using mass media, newsletters, personal invitations, emails, and website  - Documentation is an important issue and the process should be standardized and it needs commitment  - Opinions of stakeholders should be disclosed since there is conflicting priorities and competing needs |
| **Information & Intelligence**  **Information & Intelligence** | - Institutional capacity  - Mechanisms to check sources of funding  - Dissemination of information  - Generation of Information  - Stakeholders relationships  - Evidence-based approach  - Other type of information used  - Factors affecting use of information | **GAPS**  - There is **no** allocation of budget for research  - MoH has **no** specialized unit that deal with research analysis for policymaking nor make raw data generated at service delivery level accessible to researchers  - There is **no** mechanism in place to check sources of funding nor validate data sources    - Progress reports and M&E reports are generated but **not** published and are only shared with some stakeholders by emails |
|  |  | **STRENGTHES**  - MoH use data collection tools like registries, surveys and other statistics and data management tools  - MoH collaborates with research centres in the country  - All staff are qualified to access and use research based on their technical background (all are Master degree holders)  - The strategy was informed by scientific evidence that is up to date, reliable and of good quality, comprehensive and locally appropriate  - Other type of information that influenced the strategy include existing laws and political will and committeemen, while financial information and public opinion were not taken into consideration  - Factors that influenced the development of the mental health strategy in addition to evidence base include: availability of funding from international donors, the Syrian refugees crisis and their influx to Lebanon, interest of stakeholders to work on this strategy and the recommendations of UN agencies including WHO |
| **Responsiveness**  **Responsiveness** | - Adequate basic services and prompt attention  - Assessments for public preferences and satisfaction  - Rights & Responsibilities  - Institutional capacity  - Adequate health services  - Respect for dignity & confidentiality  - Factors that affect responsiveness of the government  - Contextual factors | **GAPS**  - The strategy does **not** explicit the benefit package to be provided, how referral will take place from one level of care to other nor it states reasonable timeframe to provide services needed  - Need assessment was **not** conducted as part of the strategy formulation process  - The strategy did **not** set that patient satisfactory surveys should be conducted  - The strategy mention only the rights of the patients but **not** their responsibilities  - There is **no** capacity for MoH to collect public needs and opinions |
|  |  | **STRENGTHES**  - The strategy ensures that all will have access to quality services including disadvantaged/vulnerable groups  - The strategy ensures that all health services related to mental health will respect the confidentiality and dignity of all  - The factors that can influence the responsiveness of the MoH to public needs include: availability of resources, political implications, security and safety of staff, the leadership from the community and the flexibility of the administration  - The Syrian crisis and the creation of a national program fasten the process to set a national strategy |
